# Pharmacogenetic inhibition of lumbosacral sensory neurons alleviates visceral hypersensitivity in a mouse model of chronic pelvic pain

**DOI:** 10.1371/journal.pone.0262769

**Published:** 2022-01-25

**Authors:** Alison Xiaoqiao Xie, Nao Iguchi, Taylor C. Clarkson, Anna P. Malykhina

**Affiliations:** Department of Surgery, School of Medicine, Anschutz Medical Campus, University of Colorado, Denver, Colorado, United States of America; Northwestern University, UNITED STATES

## Abstract

The study investigated the cellular and molecular mechanisms in the peripheral nervous system (PNS) underlying the symptoms of urologic chronic pelvic pain syndrome (UCPPS) in mice. This work also aimed to test the feasibility of reversing peripheral sensitization *in vivo* in alleviating UCPPS symptoms. Intravesical instillation of vascular endothelial growth factor A (VEGF_A_) was used to induce UCPPS-like symptoms in mice. Spontaneous voiding spot assays and manual Von Frey tests were used to evaluate the severity of lower urinary tract symptoms (LUTS) and visceral hypersensitivity in VEGF_A_-instilled mice. Bladder smooth muscle strip contractility recordings (BSMSC) were used to identify the potential changes in myogenic and neurogenic detrusor muscle contractility at the tissue-level. Quantitative real-time PCR (qPCR) and fluorescent immunohistochemistry were performed to compare the expression levels of VEGF receptors and nociceptors in lumbosacral dorsal root ganglia (DRG) between VEGF_A_-instilled mice and saline-instilled controls. To manipulate primary afferent activity, Gi-coupled *D*esigner *R*eceptors *E*xclusively *A*ctivated by *D*esigner *D*rugs (Gi-DREADD) were expressed in lumbosacral DRG neurons of *TRPV1-Cre-ZGreen* mice via targeted adeno-associated viral vector (AAVs) injections. A small molecule agonist of Gi-DREADD, clozapine-N-oxide (CNO), was injected into the peritoneum (*i*. *p*.) in awake animals to silence TRPV1 expressing sensory neurons *in vivo* during physiological and behavioral recordings of bladder function. Intravesical instillation of VEGF_A_ in the urinary bladders increased visceral mechanical sensitivity and enhanced RTX-sensitive detrusor contractility. Sex differences were identified in the baseline detrusor contractility responses and VEGF-induced visceral hypersensitivity. VEGF_A_ instillations in the urinary bladder led to significant increases in the mRNA and protein expression of transient receptor potential cation channel subfamily A member 1 (TRPA1) in lumbosacral DRG, whereas the expression levels of transient receptor potential cation channel subfamily V member 1 (TRPV1) and VEGF receptors (VEGFR1 and VEGFR2) remained unchanged when compared to saline-instilled animals. Importantly, the VEGF_A_-induced visceral hypersensitivity was reversed by Gi-DREADD-mediated neuronal silencing in lumbosacral sensory neurons. Activation of bladder VEGF signaling causes sensory neural plasticity and visceral hypersensitivity in mice, confirming its role of an UCPPS biomarker as identified by the *M*ultidisciplinary *A*pproach to the *S*tudy of *C*hronic *P*elvic *P*ain (MAPP) research studies. Pharmacogenetic inhibition of lumbosacral sensory neurons *in vivo* completely reversed VEGF_A_-induced pelvic hypersensitivity in mice, suggesting the strong therapeutic potential for decreasing primary afferent activity in the treatment of pain severity in UCPPS patients.

## Introduction

Urologic chronic pelvic pain syndrome (UCPPS) is characterized by chronic pain in the pelvic region or genitalia that is often accompanied by urinary frequency and urgency [1]. The recent studies from the *M*ultidisciplinary *A*pproach to the *S*tudy of *C*hronic *P*elvic *P*ain (MAPP) Research Network identified nociceptive sensitization in the central [1] and peripheral nervous systems [2] as key elements underlying visceral pain and voiding dysfunction in UCPPS. Patients with UCPPS reported higher sensitivity to pressure pain, which correlated with a lower likelihood of symptom improvement using current treatment strategies [3]. Many patients complained of widespread systemic pain, which correlated with more severe UCPPS symptoms than those with pelvic pain only [4]. In addition, longitudinal clinical changes in UCPPS were associated with structural and functional disturbances in the sensorimotor systems of the brain representing pelvic area [5]. These data suggested that increased pain sensitivity significantly contributes to UCPPS symptoms [1].

Studies in UCPPS patients have identified VEGF and VEGFR1 as potential biomarkers [6]. VEGF signaling has been shown to be crucial in vascular development during embryogenesis and angiogenesis [7]. Recently, VEGF signaling was identified in a variety of non-endothelial cells, including developing neurons [8]. Elevated VEGF has been found in the urine of UCPPS patients compared to healthy controls. Urine concentration of VEGFR1 in men and VEGF level in women were significantly higher in UCPPS patients than in healthy controls, both of which were associated with the severity of clinical symptoms [6]. In addition, increased VEGF levels have been detected in bladder biopsy samples from patients with interstitial cystitis (IC) compared to controls, with levels correlating with pain severity [9, 10]. A series of investigations using animal models revealed the key role of VEGF signaling in bladder inflammation-induced voiding dysfunction [11–13]. In addition, VEGF signaling was implied in neuropathic [14] and cancer [15] pain. Taken together, these data strongly suggest an important role of VEGF signaling in target organs/tissue in developing nociceptive sensitization. In this study, we utilized a VEGF_A_-induced mouse model of UCPPS [16] to study the potential mechanisms linking VEGF_A_ upregulation in the urinary bladder and visceral hypersensitivity in mice. We also tested the feasibility of normalizing pelvic visceral sensitivity by silencing lumbosacral sensory neurons *in vivo* using pharmacogenetic inhibition of neuronal activity.

MAPP studies also revealed important sex differences in the prevalence and severity of UCPPS symptoms. Higher prevalence of pain outside of the urinary bladder was detected among women (58.8%) than men (45.6%) [1]. In addition, while the urine levels of VEGF and VEGFR1 were both identified as potential biomarker for women with UCPPS, only VEGFR1 was associated with the severity of clinical symptoms in men [6]. In the current study, we set out to assess the potential sex differences in both bladder physiology and VEGF_A_-induced pain severity and urinary symptoms.

Overall, our study aimed to investigate the neural mechanisms underlying VEGF-dependent hypersensitivity and LUTS in mice, with emphases on 1) sex differences in VEGF-induced UCPPS symptoms; 2) the involvement of bladder VEGF signaling in regulation of myogenic vs. neurogenic responses of detrusor muscle contractility; and 3) the translational potential of alleviating visceral hypersensitivity and LUTS by inhibiting nociceptive afferent activity.

## Materials and methods

### Animals

The study was conducted on 59 adult mice (4 to 6 months, 29 males and 30 females) on C57BL6/J (B6) background. Balanced number of age-matched males and females was used in each group. Experimental data were compared between the sexes first, and then combined when no significant sex differences were detected.

The study used the following two strains of mice: B6.129-Trpv1tm1(cre)Bbm/J (TRPV1-Cre, Jackson Laboratory, Stock No: 017769) and B6.Cg-Gt(ROSA)26Sortm6(CAG-ZsGreen1)Hze/J. All transgenic mice were purchased from Jackson Laboratory (Bar Harbor, ME) and maintained on B6 background by University of Colorado Anschutz Medical Campus (CU-AMC) breeding core (Aurora, CO). All genotyping was performed by Transnetyx (Cordova, TN). Mice were housed in a temperature-controlled environment at the CU-AMC vivarium on a 14-hour light/10-hour dark cycle, with *ad libitum* access to food and water. All experiments took place during the light cycle. All animal procedures were performed at the CU-AMC according to the protocols approved by the IACUC of CU-AMC (No. 00571).

Particularly, TRPV1-Cre mice were used to enable selective pharmacogenetic inhibition of TRPV1-expressing sensory neurons. To identify sensory neurons and nerve fibers involved in pain signaling, TRPV1-Cre mice were bred to ZsGreen mice, in which ZsGreen expression is Cre-dependent, restricted in TRPV1-expresssing cells. TRPV1-Cre^+/+^:: ZsGreen^+/+^ mice (hereafter referred to as homozygous TRPV1-Cre-ZsGreen mice) were maintained on homozygous breeding and age- and sex- matched homozygous ZsGreen^+/+^ mice were used as their experimental controls.

For AAV-mediated viral injection experiments, TRPV1-Cre^+/+^ mice were first backcrossed to C57BL6/J (Jackson Laboratory, #000664), then the TRPV1-Cre^+/-^ mice were bred to ZsGreen^+/+^ mice. 50% of the offspring were TRPV1-Cre^+/-^:: ZsGreen^+/-^ (hereafter referred to as TRPV1-Cre-ZsGreen mice) and 50% of the offspring were ZsGreen^+/-^ (littermate controls).

### Intravesical instillation of VEGF_A_ and experimental timeline

**Fig 1:**
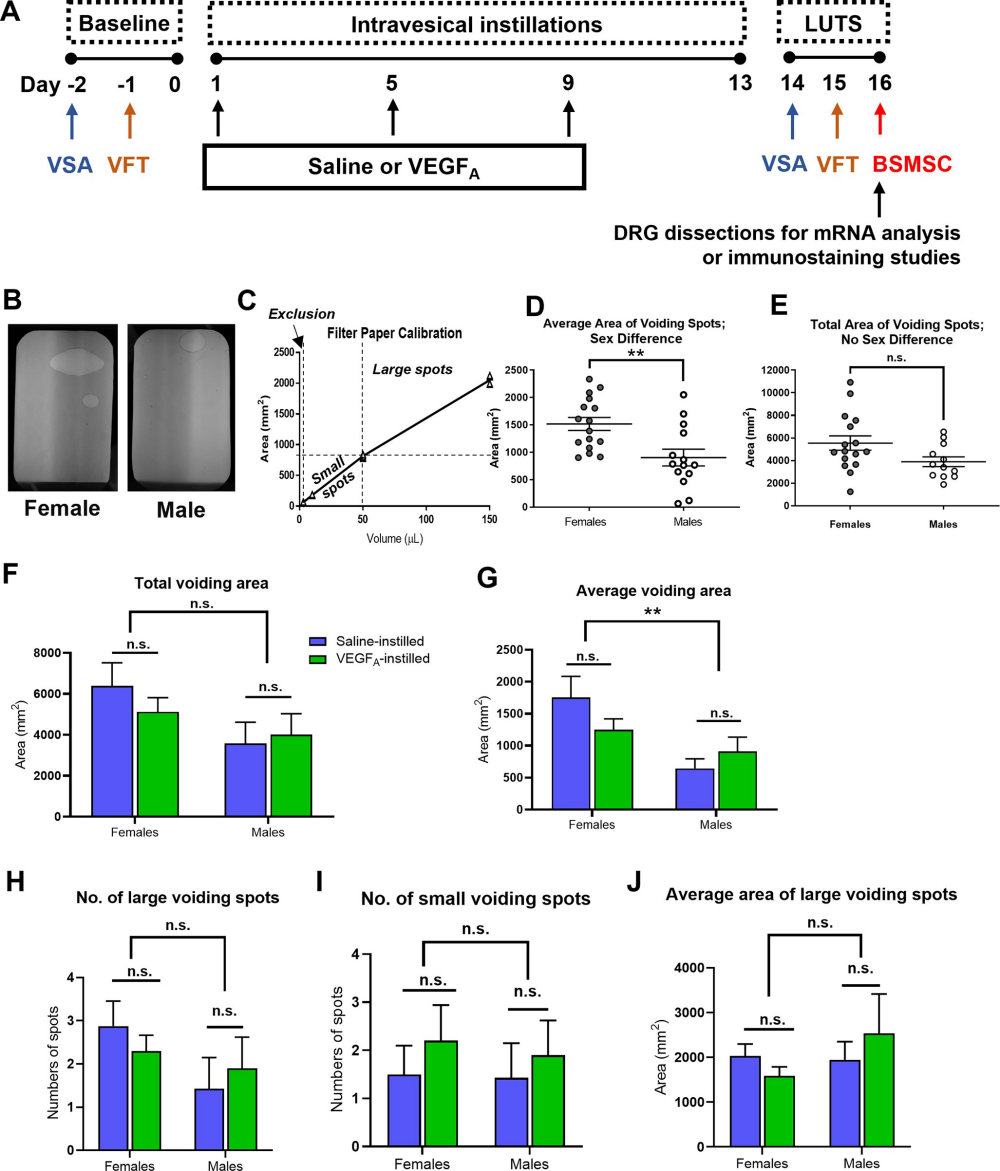
Intravesical instillation of VEGF_A_ did not affect spontaneous voiding behavior in awake mice. (A). Experimental timeline. Voiding spot assays (VSA) and Von Frey tests (VFT) were performed before (baseline) and after the instillations. Bladder smooth muscle strip contractility (BSMSC) recordings were performed at the end of the study. (B). Representative images of voiding patterns in female (left) and male (right) TRPV1-Cre-ZsGreen mice before instillations (baseline). (C). The “area-to-volume” standard curve in VSA. (D). Sex differences were detected in the average area of voiding spots (the average volume of each voiding) at baseline. (E). No sex differences were identified in the total area of voiding spots (total volume of voiding) at the baseline. (F). There were no significant differences in the total voiding area between mice that received saline and those that received VEGF_A_ intravesical instillation. (G). There were no significant differences in the average voiding area between mice that received saline and VEGF_A_ intravesical instillation. (H-J). No VEGF_A_-induced differences were found in the numbers of large (H) and small (I) voiding spots. (J) The average area of large voiding spots was not different between saline- and VEGF_A_-instilled groups.

Intravesical instillations of mouse recombinant VEGF, VEGF_A_ (also known as VEGF_165_) were performed to induce transient bladder inflammation and subsequent pelvic hypersensitivity, as previously described [16]. VEGF_A_ was purchased from ProSpec-Tany TechnoGene Ltd, (Rehovot, Israel; catalog #cyt-336 or MGC70609) and diluted in saline (6.41 nM in 100 μL). TRPV1-Cre-ZsGreen mice and ZsGreen mice were anesthetized with isoflurane (1.5%) and transurethrally catheterized. Female mice were catheterized with sterile 24-gauge BD Insyte-N Autoguard polypropylene catheter (Becton Dicknson, Sandy, Utah; REF 381411). Male mice were catheterized with sterile BD INTRAMEDIC Polyethylene Tubing/PE50 (Becton Dicknson, Sandy, Utah; REF 63019–048) [17]. All animals received three 100 uL instillations of VEGF_A_ or saline during the two-week of induction period [17], as described in Fig 1. Intravesical instillations were performed via urethral catheter with a syringe attached to one end. To ensure a consistent contact of the substances with the bladder lumen, and to avoid reflux or leakage, catheters were occluded and left in place for 30 minutes.

Spontaneous voiding spot assays (VSA) and manual Von Frey tests (VFT) were performed to assess the voiding behavior and visceral mechanical sensitivity. These experiments were performed *in vivo* before (day -2 and day -1, respectively) and after (day 14–15) VEGF_A_ instillations (day 1, 5 and 9) on the same animals (Fig 1A). After *in vivo* assessments, mice are sacrificed and used either in bladder smooth muscle strip contractility recording (BSMSC) or perfused with 4% paraformaldehyde (PFA) followed by DRG extractions and immunohistochemistry (Day 16, Fig 1A). In animals received AAV injections, Von Frey tests were performed three times on the same animal: before AAV injections, ~4 weeks after AAV injections and before VEGF_A_ instillations, as well as after VEGF_A_ instillations (Figs 6C and 7B).

### Spontaneous voiding spot assays (VSA)

Clean cages equipped with elevated wire grid (1 cm above the filter paper, 0.64 cm^2^ opening) were lined with a single layer of absorbent filter paper. Group-housed mice were transferred into individual cages with *ad libitum* access to water but no food during 3-hour testing period (9 a.m. to noon). Animals were returned to group housing after the assay. No acclimation was performed prior to testing. Voiding spots were imaged under ultraviolet (UV) light (Fig 1B). Overlapping void spots were distinguished based on the brightness under UV light. The number of voiding spots of each animal was recorded, and area of each voiding spot was measured using Image J (Bethesda, MD). Voiding spots of < 62 mm^2^ (corresponding to 3 μL of urine, Fig 1C) were excluded from analysis as previously described [18]. The number of large (≥ 815 mm^2^, corresponding to ≥ 50 μL) and small (< 815 mm^2^) micturition spots on the filter paper was counted [18]. The total area of the voiding spots, the mean area of the voiding spots, and the number of large and small voiding spots were determined for each filter paper. Data generated in males and females were first compared by Student’s *t*-test. One-way ANOVA was performed to compare data between saline and VEGF_A_ groups within males or female groups. A *P* value <0.05 was considered statistically significant. All data are expressed as the mean ± standard error of the mean (SEM). Analyses and figures were conducted using Graphpad Prism 7 (San Diego, CA).

### Manual Von Frey tests (VFT)

**Fig 2:**
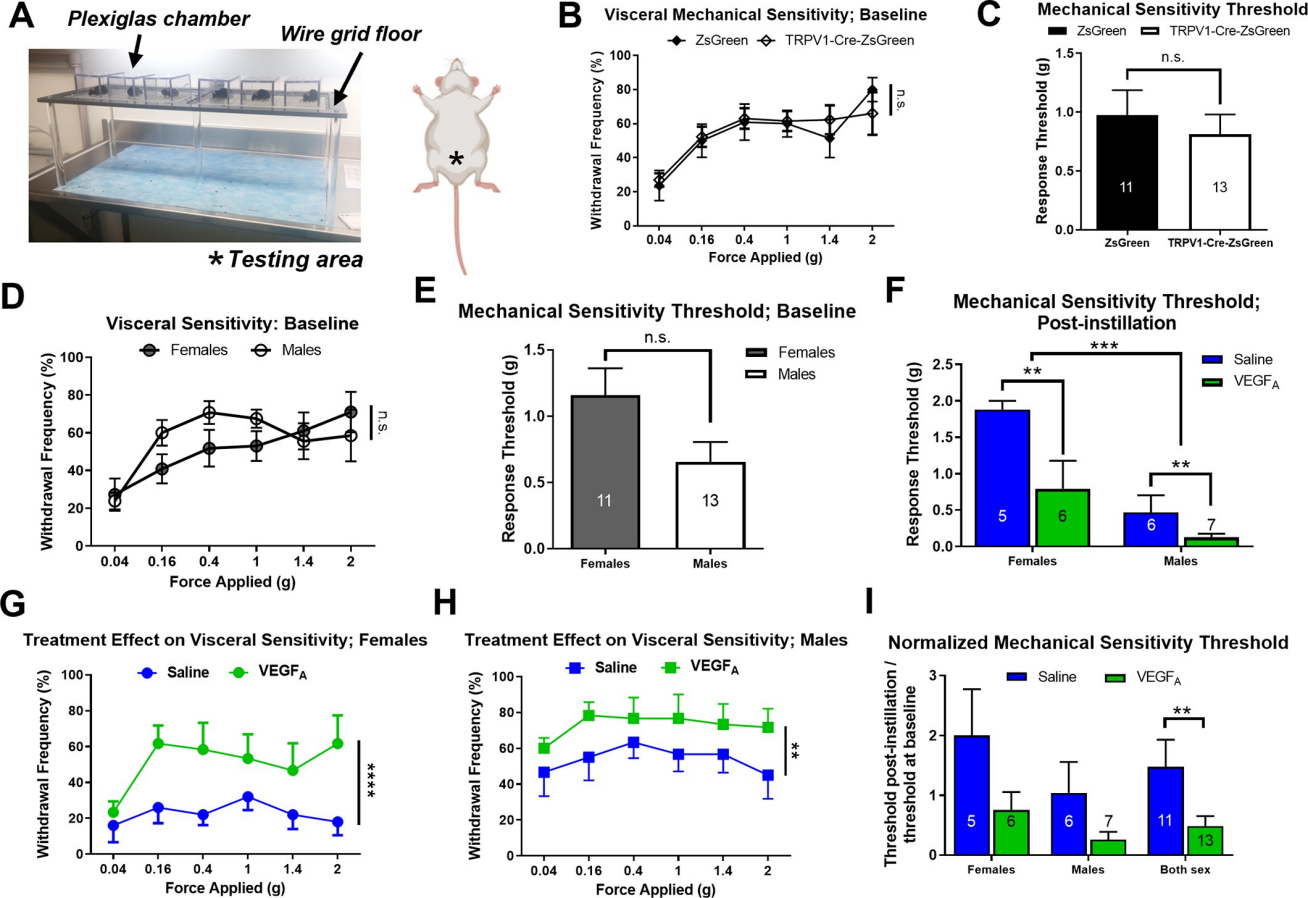
Intravesical instillation of VEGF_A_ led to visceral mechanical hypersensitivity. (A). A set up for Von Frey testing. The star indicates the pelvic area where the filaments were applied to. (B). The withdrawal “frequency-response” curve showed no significant difference in baseline visceral mechanical sensitivity between TRPV1-Cre-ZsGreen mice and ZsGreen mice. (C). No significant differences were identified in the baseline response threshold between TRPV1-Cre-ZsGreen mice and ZsGreen mice. (D-E). No sex differences were identified in the baseline visceral mechanical sensitivity. (D). The withdrawal frequency-response curve at baseline. (E). The mechanical response threshold at baseline. (F). VEGF_A_-instillation lowered mechanical response threshold in both male and female mice. Male mice showed significantly lower response threshold to visceral mechanical stimulation than females. (G). VEGF_A_-instillation significantly increased the withdrawal frequencies to visceral mechanical stimulation in female mice. (H) VEGF_A_-instillation significantly increased the withdrawal frequencies to visceral mechanical stimulation in male mice. (I) Normalized response threshold showed no sex differences, but significant VEGF_A_- induced decreases in response threshold in B6 mice. Source of the schematic of mouse underside in Fig 2A: https://biorender.com/icon/species/rodents/mouse-supine-pregnant-1/.

Mice were individually placed in clear plexiglass chambers (7.5 cm x 7.5 cm x 15 cm) equipped with wire grid floors (0.5 cm^2^ grid) (Fig 2A). Following adequate habituation (usually 1~1.5 hours, until the extinction of exploratory behavior), a series of force-generating filaments (0.04, 0.16, 0.4, 1, and 2g; Stoelting, Wood Dale, Illinois) were applied to the lower abdomen of the mice in the vicinity of the bladder (Fig 2A, insert). Retraction of the abdomen, licking or scratching the stimulated area, or jumping are considered positive responses to the stimuli. 10 repetitions were conducted with each filament and the percentage of positive responses to each force was recorded as withdrawal frequency (%). The response threshold was the force produced by the first filament that elicited more than a 50% withdrawal rate, and when over 50% withdrawal frequency was also observed in response to the next filament with higher force. In case if the response rates were always under 50%, the highest tested force (2g, ≤10% of the weight of the animals) was used as the response threshold. When the animals showed higher than 50% withdrawal frequency to all filament tested, the lowest force used (0.04g) was counted as the response threshold.

The withdrawal frequency data were analyzed using two-way ANOVA with sex/genotype/treatment and filament forces as between subjects’ factors. Response threshold data were analyzed with *t-*test. All data are expressed as the mean ± SEM. Graphpad Prism 7 was used to analyze the data and generate figures.

In experiments involving CNO injections, two Von Frey tests were performed on the same experimental animals on two consecutive days. Animals were randomly selected to receive saline or CNO on the first day of testing, then they received the other chemical on the second day of testing. Saline or CNO was administered *i*. *p*. after habituation period and 5 minutes before the testing period.

### Bladder smooth muscle strip contractility recording (BSMSC)

**Fig 3:**
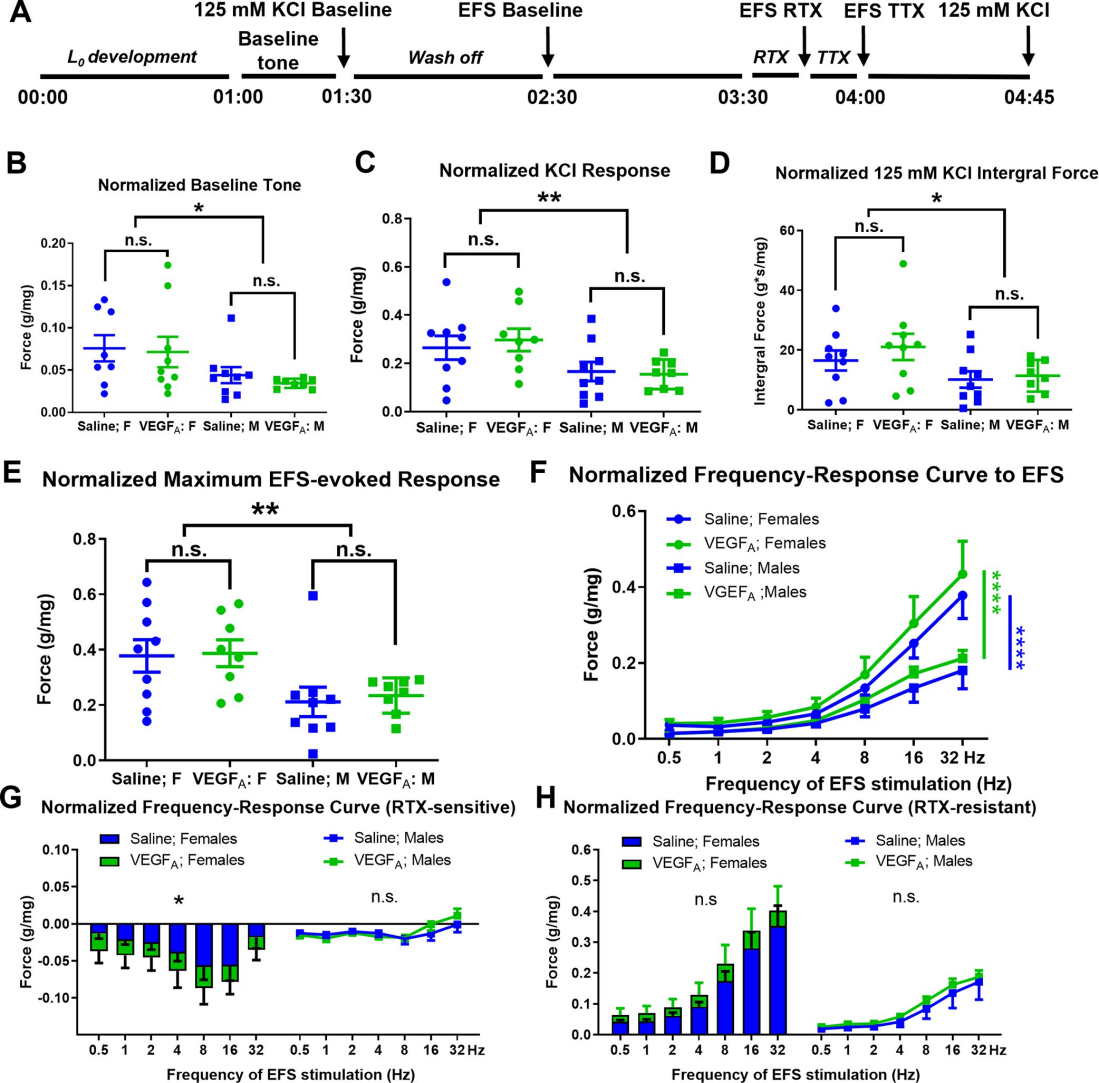
Intravesical instillation of VEGF_A_ did not affect intrinsic detrusor contractility but enhanced RTX-sensitive, nerve-mediated detrusor strip contractility in female mice. (A). Experimental timeline of BSMCS studies. L_0_ was established during the first hour of each experiment. Bladder strips were subsequently exposed to 125mM KCl, RTX, TTX, and EFSs. (B). Basal force recorded before the first KCl application. Female mice displayed higher baseline force than male mice. (C-D). Bladder muscle contractility force induced by 125 mM KCl application. (C). Female mice exhibited higher KCl-induced bladder muscle contraction than male mice. (D). The integral force of bladder muscle KCl responses. Female mice exhibited higher integral force of KCl-induced bladder muscle contractions than male mice. (E-H). Nerve-stimulation induced muscle responses. (E). Female mice showed higher maximum bladder muscle contraction induced by EFS than male mice. (F). Sex differences were also detected in the EFS-induced frequency-response curves. (G). Bladder strips isolated from VEGF_A_-instilled mice exhibited significantly greater RTX-sensitive bladder contractions when compared to those isolated from saline-instilled female animals. (H). No significant differences were detected in RTX resistant, nerve-evoked bladder contractions between saline- and VEGF_A_-instilled mice in either sex.

Mice were euthanized with CO_2_ overdose and their bladders were quickly excised above the level of urethral orifices. Each bladder was dissected longitudinally to create two bladder strips; the urothelium/lamina propria was left intact. Individual bladder strips were mounted vertically in a 10 mL organ bath with Tyrode’s solution containing (in mM): NaCl 120, KCl 6, MgCl_2_ 1.2, NaH_2_PO4 1.2, CaCl_2_ 2.5, NaHCO_3_ 14.4, and glucose 11.5, pH = 7.2) with continuous oxygenation (95% O_2_/5% CO_2_) [19]. Optimal contractile force (L_0_) was established for each bladder strip as previously described [19], followed by 30 mins of stabilization period (Fig 3A, 01:00–01:30).

High KCl solution (125mM) was applied to all bladder strips to induce maximum muscle contractility. The basal muscle tone of each muscle strip was calculated based on the spontaneous muscle contractions during the last 5 mins recording before the first KCl application (Fig 3A, 01:25–01:30). The maximum contraction forces were calculated as the peak amplitude of KCl-induced muscle contractions. KCl-induced integrated forces (g*s/mg) were also calculated. These parameters were used to represent the myogenic contractility of the detrusor strips.

After washing off the high KCl solution, electric field stimulation (EFS) in ascending order of frequencies (0.5–32 Hz) was applied to the strips to stimulate neurotransmitter release from peripheral nerve terminals located in the bladder wall [20]. The muscle contractions in response to EFSs were plotted against the stimulation frequency to generate frequency-contractility curves for each muscle strip. Resiniferatoxin (RTX; 100 nM) and Tetrodotoxin (TTX; 1 μM) were used to evaluate sensory nerve-evoked vs. all nerve-evoked muscle responses, respectively. All drugs were diluted in Tyrode’s solution, pre-oxygenated, and applied directly to the tissue bath.

At the end of experiments, KCl responses of all strips were measured again to evaluate the muscle viability. Muscle strips showing >10% change in their final KCl response compared to their KCl baseline response were excluded from further analysis. After each experiment, strip wet weights were recorded and used to calculate normalized contraction forces (g/mg) for each muscle strip. All data are expressed as the mean ± SEM. Statistical significance was assessed by one-way ANOVA followed by Fisher`s post hoc test for multiple comparisons. Graphpad Prism 7 was used to analyze the data and generate figures.

### Real-time PCR (qPCR)

Total RNA isolated from lumbosacral (L6-S2) DRG from each group of mice (N = 5 per group, 3 males, 2 females) using RNeasy Plus Micro kit (Qiagen Germantown, MD) was transcribed into cDNA, and utilized in quantitative real-time PCR (qPCR) assay, as previously described [18]. Expression levels of each gene were calculated as fold changes based on ΔΔCt values. Data were normalized to a housekeeping gene, Glyceraldehyde 3-phosphate dehydrogenase (Gapdh). All data are expressed as the mean ± SEM. Sequences of primers used for the qPCR are listed in Table 1.

**Table 1:** Primer sequences used in qPCR experiments.

Gene Name		5’-3’ Primer Sequence	Accession Number
**VEGF**_**A**_	Fw	GCACATAGAGAGAATGAGCTTCC	M95200.1
	Rv	CTCCGCTCTGAACAAGGCT	
**VEGFR1**	Fw	TAAGCCTGGGGAACTCATTCT	AK005502.1
	Rv	CCAAAGATGCGACTGTAATGCTG	
**VEGFR2**	Fw	CGAGACCATTGAAGTGACTTGCC	NM_010612.3
	Rv	TTCCTCACCCTGCGGATAGTCA	
**TRPA1**	Fw	CACAGACCG ACTAGATGAAGAAG	NM_001348288.1
	Rv	CAGGAGGATGTCAGCATTGT	
**TRPV1**	Fw	CCGGCTTTTTGGGAAGGGT	NM_001001445.2
	Rv	GAGACAGGTAGGTCCATCCAC	
**nNOS**	Fw	ACCAGCACCTTTGGCAATGGAG	NM_008712.3
	Rv	GAGACGCTGTTGAATCGGACCT	
**PKCγ**	Fw	GCACCTGAGATCATTGCCTATC	NM_011102.4
	Rv	CTGTCCTGCCAACATCTCATAC	
**TUBB3**	Fw	TAGACCCCAGCGGCAACTAT	NM_023279.3
	Rv	GTTCCAGGTTCCAAGTCCACC	

### Fluorescence immunolabeling in lumbosacral DRG

Lumbosacral L5-S2 DRG were isolated from saline- and VEGF_A_- instilled mice following 4% PFA perfusion. Samples were post-fixed, cryoprotected, then frozen in Tissue-Tek® O.C.T. Compound (Sakura Finetek USA, Inc., Torrance, CA). Thin sections (10~15 μm) were cut using a cryostat (Leica, Buffalo Grove, Illinois) and stained using standard immunohistochemistry protocol. Blocking solution contained 10% donkey serum and 0.2% Triton X-100 in phosphate-buffered saline (PBS). The primary and secondary antibodies used in this manuscript are listed in Table 2, and were previously tested (S1 Fig). All Alexa Fluor secondary antibodies (1:500) were purchased from Invitrogen (Waltham, MA). All images were taken using a Zeiss LSM780 microscope equipped with a two-photon laser system. For 488 channel, all images were taken with 0.25% laser intensity, whereas 3% laser power was used for all images taken in 594 channel. The low laser power for 488 channel was chosen due to the high intensity of ZsGreen signal. The pinhole size was set at 1 AU for each channel, and 1x digital gain and 0 digital offset were used. The master gain for 488 channel was set at 700, while the 594 channel master gain was set at 800. Three to four images were taken from three DRG tissue sections (one image per tissue section) from each animal. The intensity of fluorescence labeling was measured using Fiji (ImageJ). Graphpad Prism 7 were used to analyze the data and generate the figures. All data are expressed as the mean ± SEM.

**Table 2:** Primary and secondary antibodies used.

Antibody	Vendor	Catalog No.	Dilution
Rabbit anti-ZsGreen	Clontech Laboratories	632474	1:500
Rabbit polyclonal anti-TRPA1	St John’s Laboratory	STJ193121	1:100
Mouse monoclonal anti-TRPA1	Santa Cruz Biotechnology	sc-376495	1:50
Rabbit polyclonal anti-TRPA1	Proteintech	19124-1-AP	1:50
Mouse anti-TRPV1	Santa Cruz Biotechnology	sc-398417	1:200
Mouse anti-VEGFR1/Flt-1	R&D Systems	MAB471	1:100
Mouse anti-VEGFR2	Santa Cruz Biotechnology	sc-393163	1:100
Rabbit anti-mCherry	Rockland	600-401-P16	1:500
Rabbit anti-BLBP	Millipore	ABN14	1:500
DAPI	Invitrogen	D1306	1:300
Donkey anti-rabbit IgG (H+L) (Alexa 488 conjugated)	Invitrogen	A21206	1:500
Donkey anti-rabbit IgG (H+L) (Alexa 568 conjugated)	Invitrogen	A10042	1:500
Goat anti-rabbit IgG (H+L) (Alexa 594 conjugated)	abcam	ab150080	1:500
Donkey anti-mouse IgG (H+L) (Alexa 594 conjugated)	Invitrogen	A21203	1:500

### AAV-mediated Gi-DREADD expression

To express Gi-coupled designer receptors exclusively activated by designer drugs (DREADD) in lumbosacral sensory neurons, AAV8-hSyn-DIO-Gi-DREADD-mCherry (Addgene #44362-AAV8, hereafter referred to as Gi-DREADD vectors; 2 x 10+E11 GC/ML) was delivered in TRPV1-Cre-ZsGreen mice via intrathecal injections (3 μL/animal). Intrathecal injections were performed in anesthetized mice (2% isoflurane inhalation) on a warm heating pad. A 10 μl, 26-gauge Hamilton needle was used for a bolus injection between L5 and L6 vertebrae. The needle was kept inside for 30 seconds before removal to prevent leaking. Animals were immediately taken off isoflurane and recovered on the heating pad. The Cre presence in TRPV1-expressing cells enables the expression of Gi-DREADD-mCherry (fused) in the TRPV1-expressing neurons in the lumbosacral spinal cord and DRG (Fig 5A). As controls, AAV8-hSyn-DIO-mCherry (Addgene # 50459-AAV8, hereafter referred to as mCherry vectors) was injected in TRPV1-Cre-ZsGreen mice in the similar titer (Fig 6).

The expression of mCherry in the frozen spinal cord and DRG sections was confirmed by immunohistochemistry using antibodies against mCherry (Cat. No. 600-401-P16, Rockland, Limerick, PA). Antibodies against ZsGreen (Cat. No. 632381, Takara, Shiga, Japan) were used to identify TRPV1-expressing cells. The fluorescence immunoreactivity of mCherry and ZsGreen was visualized using the Zeiss confocal microscope.

### Pharmacogenetic manipulation of sensory neuronal excitability in vivo

To activate Gi-DREADD in lumbosacral sensory neurons *in vivo*, Clozapine-N-Oxide (CNO; Sigma, St. Louis, MO; SML2304) was dissolved in physiological saline, and administered via a single *i*. *p*. injection (2 mg/kg). In voiding assay experiments, CNO or saline was administered right before the 3-hour testing period. Each animal was evaluated twice on different testing days after receiving either saline or CNO administration. In Von Frey tests, CNO or saline was administered after adequate habituation, and five minutes before the testing period. Visceral mechanical sensitivity was then assessed within an hour post CNO administration [21]. Each animal was evaluated twice on different testing days after receiving either saline or CNO. All data are expressed as the mean ± SEM.

## Results

### Intravesical instillations of VEGF_A_ did not alter spontaneous voiding behavior in awake mice

Voiding spot assay was used in awake and free-moving animals to evaluate the spontaneous voiding behavior in saline- and VEGF_A_-instilled mice [22] (Fig 1). Voiding spot assay was first performed two days before the instillations on all animals (baseline) (Fig 1A). Urine collected from mice was pipetted on filter paper in different volumes (3, 10, 50, and 150 μL); the area of voiding spot and the urine volume appeared to be a near-linear relationship (Fig 1C). Age-matched homozygous TRPV1-Cre-ZsGreen and homozygous ZsGreen mice were used. No significant difference was detected in any of the parameters examined between the two genotypes.

Both female (N = 18) and male (N = 17) mice were used in this experiment (Fig 1B). Analysis of the area of voiding spots showed significant differences between males and females at baseline (Fig 1D, nonparametric *t-*test on 63 spots/18 females and 61 spots/17 males). Males tended to void smaller volume (Fig 1D, p = 0.0042 for average area of voiding spots per animal), while male and female voided similar total volume of urine during the 3-hour test period (Fig 1E, p = 0.1208, N = 16 for females, N = 12 for males). A small number of female (N = 2) and male (N = 5) mice did not produce any voids above 3 μL during the 3-hour testing period, a behavior commonly observed in voiding spot assays [23, 24]. All 5 non-voiding males displayed spray behavior, which was reported as marking behavior often seen in dominate males [22].

After baseline recordings of voiding spots, animals were randomly selected to receive intravesical instillations of either saline or VEGF_A_. No significant differences were found in any of the parameters between the saline- and the VEGF_A_-instilled groups at the baseline. At the end of the two-week instillations, voiding spot assay was performed again on all animals (Fig 1A). The total voiding area (Fig 1F), the average voiding area (Fig 1G), the numbers of large (Fig 1H) and small (Fig 1I) voiding spots, as well as the average area of the large voiding spots (Fig 1J) were compared between the saline-instilled groups (female: N = 8; male: N = 7) and the VEGF_A_-instilled group (female: N = 10; male: N = 10). No sex or treatment differences were identified in the total voiding area (Fig 1F, saline vs VEGF: p = 0.6728, mail vs female: P = 0.0547, interaction: P = 0.3948). Male mice exhibited significant smaller average voiding area (P = 0.0038), but no significant difference was detected in the average voiding area between saline- and VEGF_A_-instilled groups (Fig 1G, saline vs VEGF: P = 0.6103, interaction between sex and treatment: P = 0.1071). VEGF_A_-instillations did not result in significant changes in the numbers of small or large voiding spots during the three-hour testing period (Fig 1H, saline vs VEGF: P = 0.9332, male vs female: 0.1422, interaction, 0.3999; Fig 1I, saline vs VEGF: P = 0.4235, male vs female: P = 0.7987, interaction: P = 0.8753). Lastly, no significant differences were identified in the average area of large voiding spots between saline- and VEGF_A_-instilled animals (Fig 1J, saline vs VEGF: P = 0.8993, male vs female, P = 0.4482, interactions: P = 0.3708). Taken together, these data showed that repeated VEGF_A_-instillations did not significantly change spontaneous voiding behavior in both sexes of mice.

### Intravesical instillations of VEGF_A_ led to visceral hypersensitivity

Manual Von Frey tests are commonly used to assess mechanical sensitivity in animal models of pain [25] including mouse models of urological disorders [26] (Fig 2A). We have reported VEGF_A_ instillations increases withdrawal frequencies to Von Frey filaments in B6 mice [16]. In this study, we performed Von Frey tests on saline- and VEGF_A_-instilled mice before (baseline) and after bladder instillations using Von Frey filaments of similar weight as in our previous study [16] (Fig 1A). Homozygous TRPV1-Cre-ZsGreen (N = 13) and homozygous ZsGreen mice (N = 11) were used in this experiment. The response frequencies were plotted as withdrawal frequency-response curve (Fig 2B). The higher the withdrawal frequency was, the more sensitive the animal was to mechanical stimulation. The minimal force to produce at least 50% of responses was identified for each animal as its response threshold (Fig 2C). Lower response thresholds indicated that the animal was more sensitive to mechanical stimulation. No significant behavioral differences in baseline visceral mechanical sensitivity were detected between TRPV-1-Cre-ZsGreen mice and ZsGreen mice (Fig 2B, frequency-response curve, TRPV-1-Cre-ZsGreen vs ZsGreen: p = 0.8898, forces: p<0.0001, interaction: p = 0.8346; Fig 2C, response threshold, p = 0.6541). In addition, no sex differences were identified in either the frequency-response curve (Fig 2D; sex: p = 0.2739, force: p<0.0001, interaction: p = 0.2300) or in the response threshold (Fig 2E; p = 0.2450) (females, N = 11; males, N = 13) at the baseline (before instillations).

After intravesical instillations, VEGF_A_-instilled mice (N = 13) showed a significantly lower response threshold when compared to saline-instilled littermate controls (N = 11) (Fig 2F; VEGF_A_ vs. saline: p = 0.0065). These data confirmed that repeated VEGF_A_ instillations increased visceral sensitivity in mice. Male mice also exhibited lower response threshold (higher visceral mechanical sensitivity) compared to female mice in both instillation groups (Fig 2F; male vs. female: p = 0.0003). This could be due to more invasive nature of urethral catheterization in males. VEGF_A_-instillation led to significant increases in the withdrawal frequencies in both female (Fig 2G; p<0.0001) and male (Fig 2H; p = 0.0040) mice when compared to their saline-instilled littermates, suggesting that VEGF-induced visceral hypersensitivity is a shared phenotype between sexes. Lastly, we normalized the response threshold after instillations in each animal to their own response threshold recorded before instillations (post-instillation value divided by pre-instillation value). VEGF-induced visceral mechanical hypersensitivity persisted (Fig 2I; p = 0.0044), suggesting that repeated exposures of VEGF in the urinary bladder induced visceral mechanical allodynia and hyperalgesia in treated mice.

### Intravesical instillations of VEGF_A_ did not change intrinsic detrusor contractility

We previously observed that intravesical instillations of VEGF_A_ led to increased density of TRPV1 positive nerve terminals in all layers of the mouse bladder [16]. Here, we hypothesized that repeated VEGF_A_ instillations into the urinary bladder may increase sensory nerve-dependent detrusor muscle contractions. To test this, bladder muscle contractility was recorded in isolated bladder strips *in situ* [20] (Fig 3A). Muscle strips were prepared from homozygous TRPV1-Cre-ZsGreen (n = 17, N = 9) and homozygous ZsGreen mice (n = 17, N = 9) that received either saline- or VEGF_A_-instillations. Both sexes (male: n = 17, N = 9; female: n = 17, N = 9) were used in this study.

The basal muscle tone of each bladder strip was recorded to reflect the baseline activity of bladder strips. Muscle strips isolated from female mice (saline group: n = 8; VEGF_A_ group: n = 9) displayed a significantly higher basal muscle tone (Fig 3B, p = 0.0132) compared to those isolated from males (saline group: n = 9; VEGF_A_ group: n = 8). However, VEGF_A_-instillations did not lead to any changes in the normalized baseline forces (p = 0.3415), suggesting that repeated VEGF_A_ exposure to the bladder lumen did not lead to changes in the basal tone of detrusor muscle.

All bladder strips were exposed to 125 mM KCl to induce maximum contractions of the muscle. The maximum contractility force was identified as the peak amplitude of KCl-induced contraction for each bladder strip [19]. Strips isolated from female mice displayed significantly higher peak amplitude (Fig 3C, p = 0.0067) and integral force (Fig 3D, p = 0.0218) of contractions in response to 125 mM KCl application. There were no significant differences in the peak amplitude (p = 0.7987) or the integrated force (p = 0.3885) of KCl responses between bladder strips isolated from saline- and VEGF_A_-instilled mice. Together, the lack of VEGF_A_-induced changes in basal muscle tone and maximum contractility suggested that intrinsic contractility of the detrusor muscle in VEGF_A_-instilled bladders remained unaffected.

### VEGF_A_ bladder instillations increased RTX-sensitive, nerve-evoked detrusor strip contractility in female mice

Next, we examined if VEGF_A_ instillations led to the changes in nerve-evoked detrusor muscle contractions. Significant sex differences were identified in the peak contractility in response to EFS stimulation (Fig 3E, p = 0.0027). However, no differences were found between bladder strips isolated from saline- and VEGF_A_-instilled animals (Fig 3E, p = 0.4020). Similarly, VEGF_A_ instillations did not lead to significant changes in the frequency response curves in either female (Fig 3F, p = 0.1974) or male bladder strips (p = 0.1739) in response to EFS. These data suggested that repeated VEGF exposure to the urinary bladder did not affect the detrusor response to neurotransmitter release from all nerve terminals in the bladder wall.

Following the evaluation of nerve-evoked bladder strip contractions, sensory- and motor- components of nerve-evoked contractility responses were assessed using RTX (100 nM) and TTX (1 μM) (Fig 3G and 3H). At the concentration used, RTX desensitizes the sensory nerve terminals in the bladder wall and, thereby, inhibits release of neurotransmitters from sensory nerve terminals [27], whereas TTX blocks all nerve endings’ ability of releasing neurotransmitters. Bladder strips were incubated in RTX-containing solutions for 15 minutes, then EFS was applied to all bladder strips (post-RTX). RTX-sensitive contractility was calculated by subtracting the baseline contraction forces to all nerve stimulations from the post-RTX contraction forces, resulting in negative values (Fig 3G). VEGF_A_ instillations significantly augmented RTX-sensitive muscle contractility in bladder strips isolated from female mice (Fig 3H left, p = 0.0114) but not in those isolated from male mice (Fig 3H right, p = 0.6284). These data suggest that intravesical VEGF instillations induce changes in bladder contractility predominantly via afferent innervation with more pronounced responses in female mice.

TTX was then applied to the bladder strips to block all nerve-induced contractile responses (Fig 3A). RTX remained in the solution, and there was no wash-off period of RTX in this experiment. EFS was applied to all muscle strips after TTX/RTX incubation and did not evoke any muscle contractions. The differences in EFS-induced muscle responses between pre- and post-TTX incubation were identified as RTX-insensitive, efferent nerve-dependent detrusor contractile responses (Fig 3H). No significant differences were found in the RTX-insensitive muscle contractions between bladder strips isolated from saline- and VEGF_A_-instilled animals in either females (p = 0.0921) or males (p = 0.3020), suggesting that the VEGF-induced neurogenic changes in detrusor contractility are mostly due to VEGF effects on afferent bladder innervation.

### Bladder VEGF_A_ instillations caused the changes in mRNA expression of TRPA1 and nNOS in lumbosacral DRG

**Fig 4:**
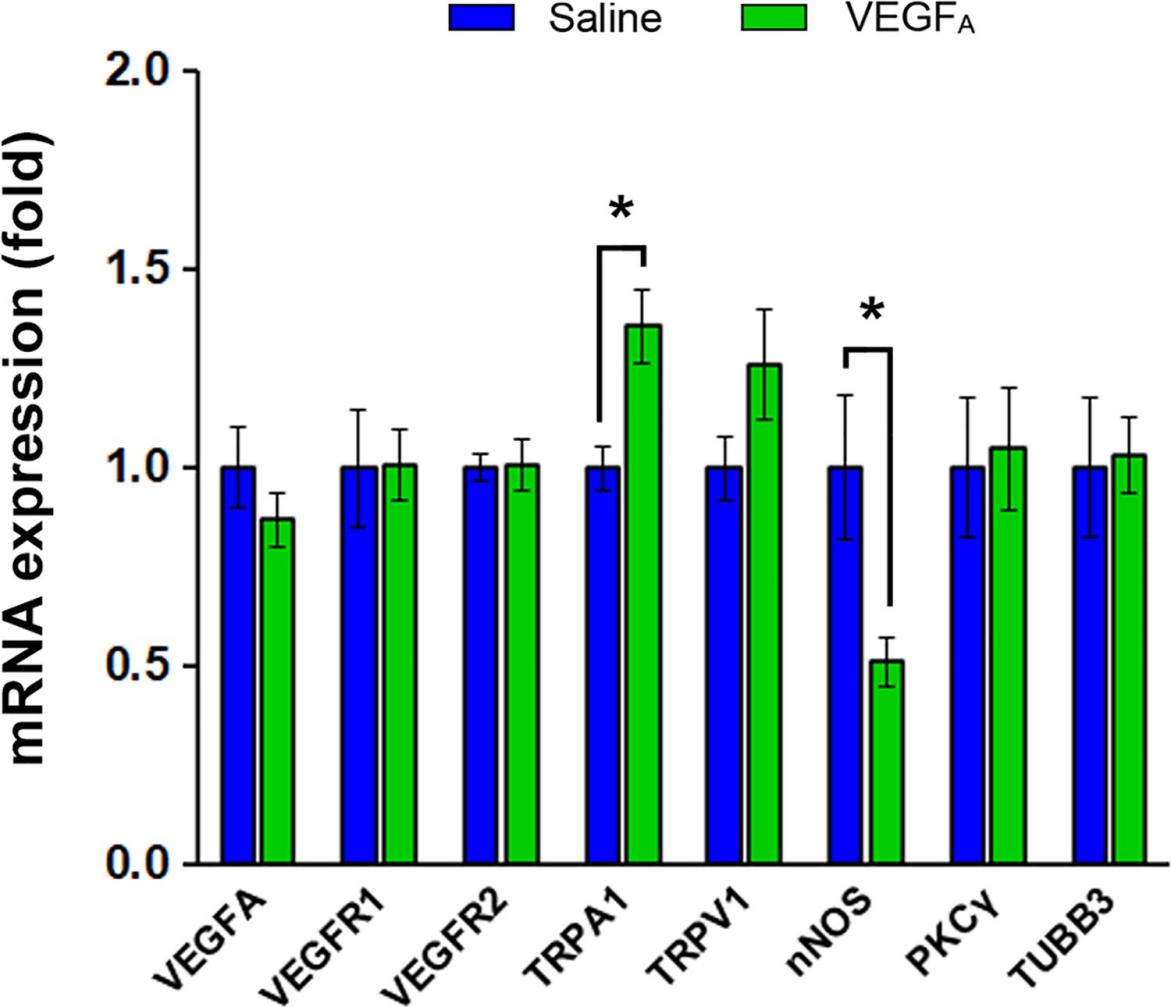
Bladder VEGF_A_ instillations caused changes in mRNA expression in TRPA1 and nNOS genes in lumbosacral DRG. Significantly higher TRPA1 mRNA expression and lower nNOS mRNA expression were detected in lumbosacral DRG isolated from VEGF_A_-instilled mice when compared to saline-instilled mice. No differences were detected in the gene expression of either VEGF receptors.

To investigate the molecular link between VEGF signaling and nociceptive sensitization, we analyzed the mRNA expression of key molecules in VEGF signaling pathways as well as nociceptors in lumbosacral DRG isolated from saline- and VEGF_A_-instilled mice (Fig 4) using primer sequences listed in Table 1. On day 16 after the first instillation (Fig 1A), lumbosacral DRG were isolated from saline- and VEGF_A_-instilled mice (N = 4 and N = 5, respectively). Significantly higher mRNA expression of TRPA1 channel (p = 0.01) and significantly lower mRNA expression of nNOS (p = 0.02) were observed in the lumbosacral DRG isolated from VEGF-instilled animals compared to those from saline-instilled animals (Fig 4). No differences in VEGFR1 or VEGFR2 mRNA were detected between lumbosacral DRG isolated from saline- and VEGF_A_-instilled mice. These data suggest an up-regulation of TRPA1 channels in lumbosacral DRG neurons following VEGF_A_ instillations in the urinary bladders.

### Bladder VEGF_A_ instillations did not affect protein expression of VEGF receptors in lumbosacral DRG

**Fig 5:**
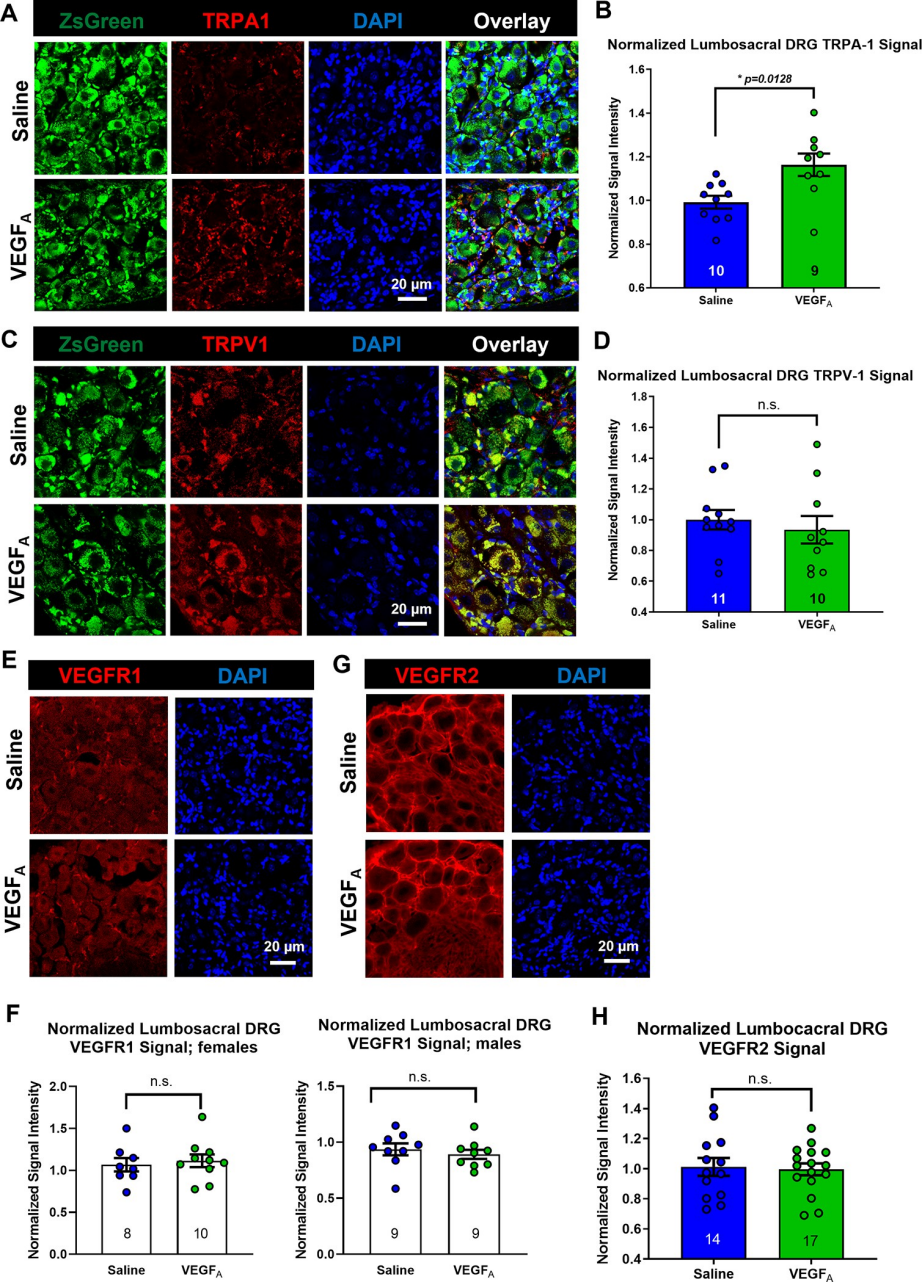
Bladder VEGF_A_ instillation led to an up-regulation of TRPA1 in lumbosacral DRG. (A). Representative data of immunofluorescence labeling of TRPA1 channel in lumbosacral DRG. Scale bar: 20 μm. (B). Normalized signal intensity of TRPA1 antibody in lumbosacral DRG isolated from VEGF_A_-instilled animals is significantly higher than in ganglia from saline-instilled animals. (C). Representative data of immunofluorescence labeling of TRPV1 channel in lumbosacral DRG. Scale bar: 20 μm. (D). Normalized signal intensity of anti-TRPV1 is not significantly different in lumbosacral DRG isolated from VEGF_A_-instilled animals when compared to those from saline-instilled animals. (E). Representative data of immunofluorescence labeling of VEGFR1 in lumbosacral DRG. Scale bar: 20 μm. (F). Normalized signal intensity of anti-VEGFR1 in lumbosacral DRG isolated from female (left) and male (right) mice. No significant difference in anti-VEGFR1 signal was detected between saline- and VEGF_A_-instilled animals. (G). Representative data of immunofluorescence labeling of VEGFR2 in lumbosacral DRG. Scale bar: 20 μm. (H). No significant difference in anti-VEGFR2 signal was detected between saline- and VEGF_A_-instilled animals.

We next performed immunofluorescent labeling of TRPA1 channel using frozen sections of lumbosacral DRG, where the somata of sensory nerves reside (Fig 5). Lumbosacral DRG were isolated from saline- and VEGF_A_-instilled mice (saline: N = 18, VEGF: N = 18) on the day 16 after the first instillation (Fig 1A). TRPA1 immunoreactivity was largely found in small-to-medium size sensory neurons and satellite glial cells in the sensory ganglia (Figs 5A and S1–S3). No significant sex difference in TRPA1 expression was detected in lumbosacral DRG (p = 0.5905). Consistent with our qPCR data (Fig 4), TRPA1 immunoreactivity was significantly higher in the lumbosacral DRG isolated from VEGF-instilled animals compared to those from the saline group (Fig 5B; saline: N = 10; VEGF: N = 9; p = 0.0118), suggesting that an upregulation of TRPA-1 channel could potentially underlie VEGF-induced visceral mechanical hypersensitivity.

Previous reports showed that bladder VEGF_A_ instillations increased TRPV1 immunoreactivity in the bladder wall [16]. In this study, we tested whether TRPV1 expression was upregulated in lumbosacral DRG following bladder instillations of VEGF_A_. Consistent with our qPCR data, the intensity of anti-TRPV1 signal was not significantly different between lumbosacral DRG isolated from saline- and VEGF_A_-instilled mice (Fig 5C and 5D; saline: N = 11; VEGF: N = 10; p = 0.5804). No significant sex difference was detected in TRPV1 signal intensity.

Also consistent with the qPCR data, no significant differences were found in the intensity of anti-VEGFR1 or anti-VEGFR2 signals in lumbosacral DRG between saline- and VEGF_A_-instilled animals. VEGFR1 immunoreactivity was primarily detected in the somata of sensory neurons and satellite glial cells (Fig 5E). Bladder VEGF_A_ instillations did not lead to detectable differences in anti-VEGFR1 signal intensity in lumbosacral DRG between saline-and VEGF_A_-instilled groups (Fig 5F; saline: N = 17; VEGF: N = 19; p = 0.9804). However, significant sex differences were found in VEGFR1 expression in DRG (Fig 5F left: females, N = 18; right: males, N = 18; p = 0.0103). In contrast, VEGFR2 immunoreactivity was not found in sensory neurons or satellite glial cells (Fig 5G). Our data showed that VEGFR2 receptors were primarily localized throughout the extensive DRG capillary network, consistent with other published studies using CD31 and VEGFR2 labeling [28]. No sex or VEGF_A_-induced differences were observed in lumbosacral sensory ganglia for VEGFR2 signal (Fig 5H; saline: N = 14, VEGF: N = 17, p = 0.8194).

### Pharmacogenetic inhibition of lumbosacral sensory neurons alleviates VEGF-induced pelvic hypersensitivity in vivo

**Fig 6:**
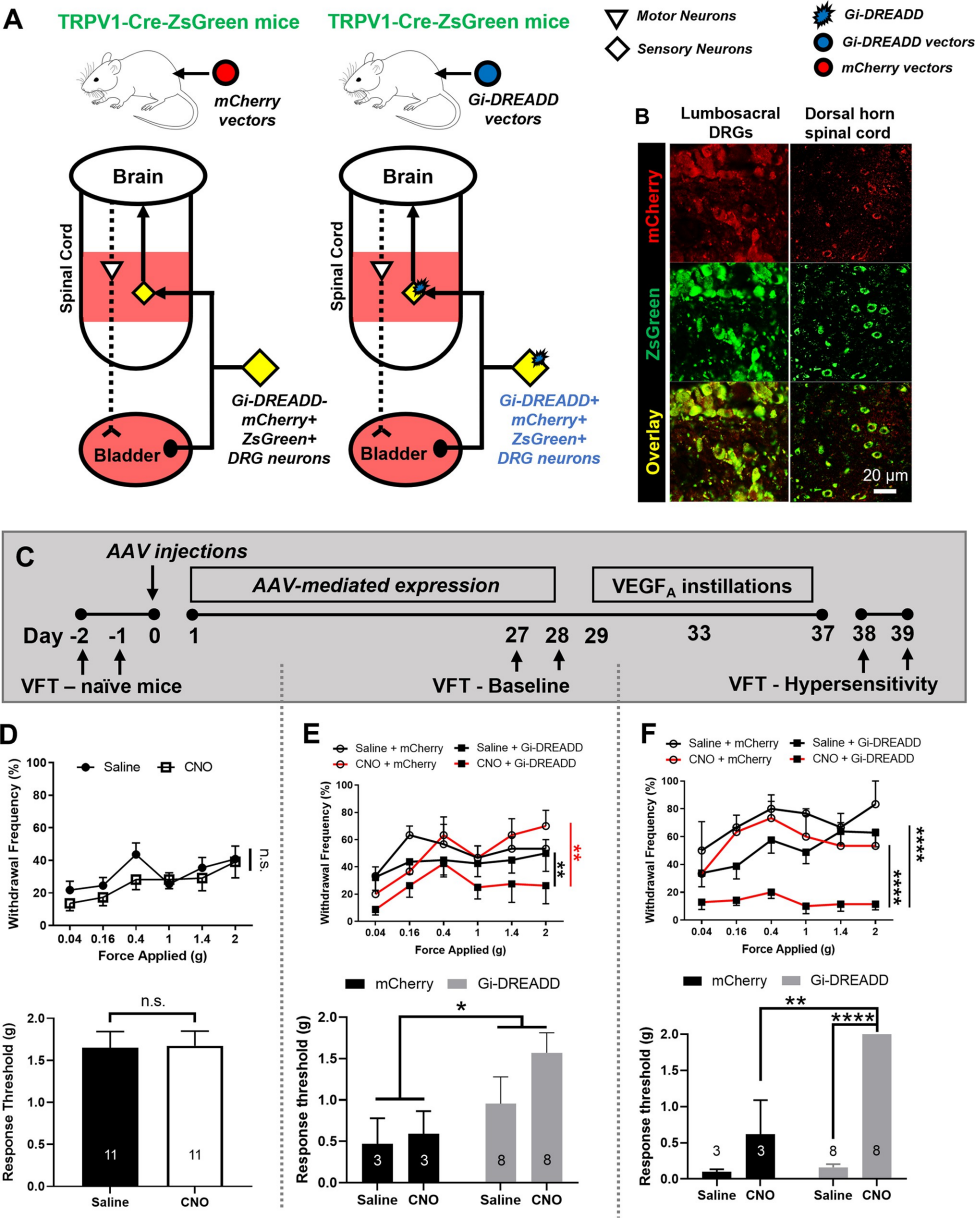
Pharmacogenetic inhibition of lumbosacral DRG neurons via AAV-mediated Gi-DREADD expression led to visceral mechanical analgesia. (A). AAV vectors containing Gi-DREADD or mCherry reporter were injected into TRPV1-Cre-ZsGreen mice to express Gi-DREADD in lumbosacral sensory neurons. (B). AAV-mediated mCherry expression in majority of sensory neurons in lumbosacral DRG. (C). Experimental timeline. Visceral mechanical sensitivity was recorded from all animals before AAV injection, after AAV injections, and then after VEGF_A_ instillations. (D). CNO did not cause changes in the frequency-response curve or response threshold of visceral mechanical sensitivity recorded from naïve animals before AAV injections. (E). When Von Frey test was performed after AAV injections, CNO significantly lowered the withdrawal frequencies and increased the response threshold in Gi-DREADD vector-injected animals but not in mCherry vector-injected animals. (F). Von Frey test performed after intravesical instillations of VEGF_A_. CNO significantly lowered the withdrawal frequencies and increased the response threshold in Gi-DREADD vector-injected animals but not in animals injected with mCherry vectors.

Collectively, our data suggested that repeated VEGF exposure to the bladder lumen induced nociceptive sensitization in lumbosacral DRG. Next, we tested the hypothesis that sensory neuronal inhibition in lumbosacral DRG may reverse VEGF-induced visceral hypersensitivity. To manipulate sensory neuronal activity *in vivo* and in awake animals, Gi-DREADD, an engineered Gi-coupled GPCR (G protein-coupled receptors) was expressed in TRPV1-expressing sensory neurons in the lumbosacral DRG (Fig 6A). Previous tracing experiments showed that hypogastric and pelvic afferents projecting to the urinary bladder arise from L1-L2 and L6-S2 lumbosacral DRG, respectively [29]. A single *i*. *p*. injection of CNO in awake animals can induce Gi-DREADD activation and hyperpolarization of Gi-DREADD-expressing neurons, which decreases the excitability of affected neurons [30]. In our case, we speculated that CNO injections would significantly decrease the excitability of TRPV1 positive, Gi-DREADD-expressing neurons in the lumbosacral spinal cord and DRG.

To express Gi-DREADD in lumbosacral primary sensory neurons, AAV8-hSyn-DIO-Gi-DREADD-mCherry (Gi-DREADD vectors) were injected *i*.*t*. into heterozygous TRPV1-Cre-ZsGreen mice (N = 8), in which Cre recombinase as well as ZsGreen reporter were expressed in the majorities of sensory neurons and satellite glial cells (Fig 6A). A small number (N = 3) of heterozygous TRPV1-Cre-ZsGreen mice received AAV8-hSyn-DIO-mCherry (mCherry vectors) injections, and served as control group. AAV8-hSyn-DIO-Gi-DREADD-mCherry injections in TRPV1-Cre-ZsGreen mice resulted in mCherry expression in majority of ZsGreen-positive neurons in lumbosacral DRG (Fig 6B). A small number of animals (N = 5, all males) was subjected to voiding spot assays before and after Gi-DREADD vector injections to test if intrathecal injections or AAV-mediated cell transduction affected spontaneous voiding behavior. No significant differences were detected in the average numbers of voiding spots, the average area of voiding spots, or the frequency-response curve to mechanical stimuli recorded four weeks after AAV injections (when Gi-DREADD/mCherry expression were expected) when compared to recordings from the same animals before AAV injections. Together, these data demonstrated our ability to effectively and selectively express Gi-DREADD in lumbosacral sensory neurons via targeted AAV injections without significant effects on the urinary function or voiding behavior.

As shown in Fig 6C, all animals that received Gi-DREADD vector or mCherry vector injections were subjected to Von Frey tests before (naïve mice, Fig 6D) and at four weeks after AAV injections (baseline, Fig 6E). CNO administration did not result in any significant differences in visceral mechanical sensitivity in naïve animals (Fig 6D, upper panel, treatment factor: p = 0.4216; lower panel, p>0.9999; N = 11 total, paired *t*-test), suggesting that CNO injections did not have “off-target” effects on pelvic sensitivity *in vivo*. Following AAV-mediated Gi-DREADD expression, CNO administration significantly reduced the baseline visceral mechanical sensitivity in mice with Gi-DREADD vectors, but not in mice with mCherry vectors (Fig 6E). This set of data suggested that Gi-DREADD activation in lumbosacral sensory neurons decreased visceral mechanical sensitivity. As shown in the upper panel of Fig 6E, CNO injections in mice expressing Gi-DREADD (N = 8) showed decreased withdrawal frequencies to all applied forces when compared to the same mice with saline administration (p = 0.0048) or mice received mCherry vectors and CNO (N = 3, p = 0.0019). CNO administration did not lead to substantial changes in mCherry vector injected mice (p = 0.8476). Furthermore, significant differences were detected in the response threshold between mice with Gi-DREADD and mCherry vector injections (p = 0.0353). However, no significant difference was detected when directly comparing Gi-DREADD-expressing mice and mCherry-expressing mice following CNO administration (p = 0.1333, unpaired *t-*test). In addition, no significant difference was detected between saline- and CNO- administration in Gi-DREADD-expressing mice (p = 0.2330, paired *t-*test) (Fig 6E, lower panel).

Subsequently, all animals were subjected to VEGF_A_ instillations, and Von Frey tests were performed on day 15 after the first instillation to assess the effects of CNO in reversing visceral hypersensitivity (Fig 6F). CNO administration did not cause any changes in the withdrawal frequencies of mCherry vector-injected animals (p = 0.0833), whereas CNO administration significantly lowered the withdrawal frequencies in mice injected with Gi-DREADD vectors when compared to the same animals after saline administration (p<0.0001) or when compared to mCherry vector-injected animals after CNO administration (p<0.0001) (Fig 6F, upper panel). CNO administration significantly increased the response threshold in Gi-DREADD vector-injected mice when compared to mCherry vector-injected mice (Fig 6F, lower panel; p = 0.0099, unpaired *t-*test) or the same mice after saline administration (p = 0.0009, paired *t-*test). Two-way ANOVA analysis showed significant interaction (p = 0.0012) with <0.0001 treatment factor (CNO vs. saline) and 0.0006 for different AAV vectors (Gi-DREADD vs. mCherry).

**Fig 7:**
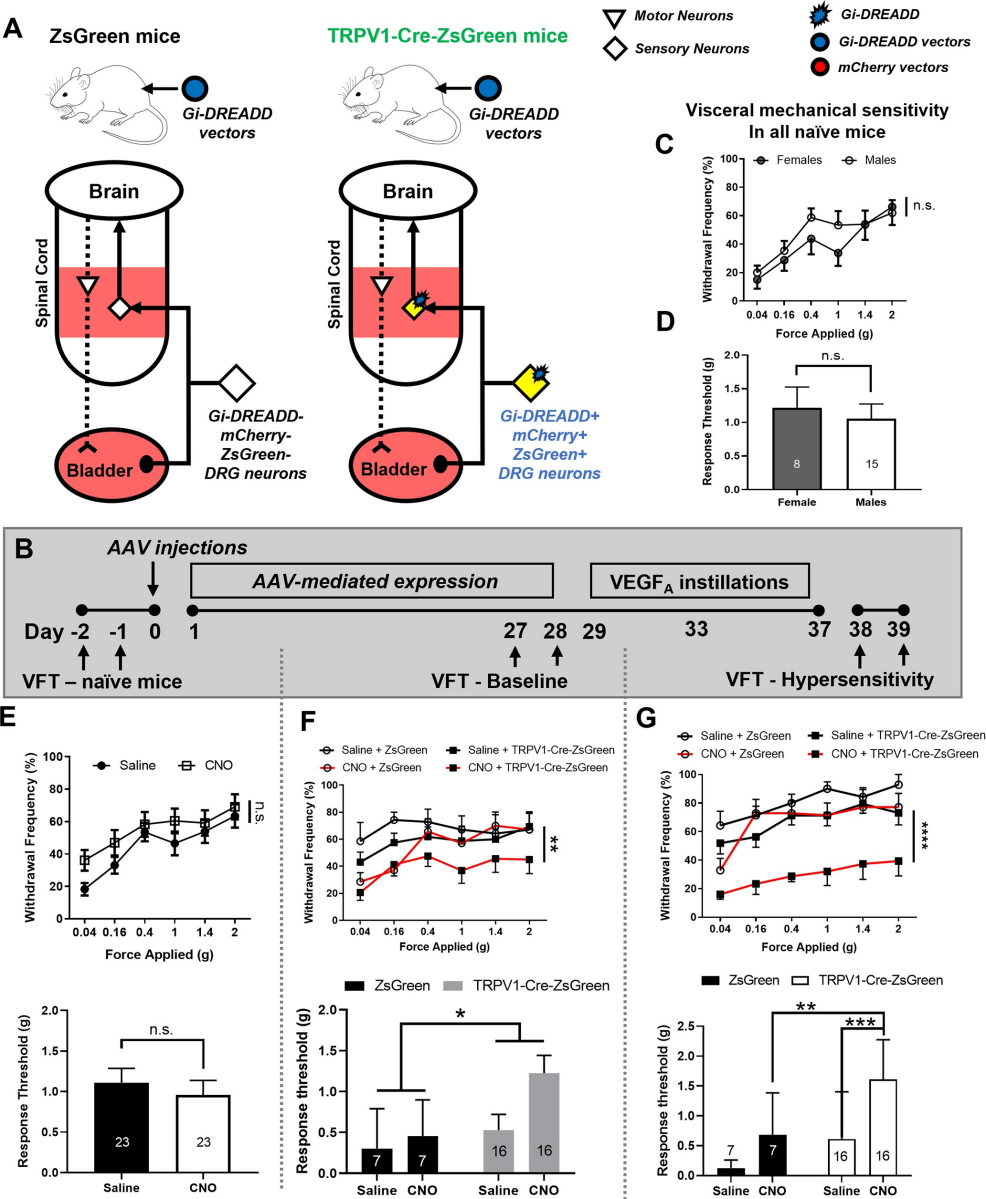
Pharmacogenetic inhibition of lumbosacral sensory neurons alleviated VEGF-induced visceral hypersensitivity in vivo. (A). AAV vectors containing Gi-DREADD were injected into TRPV1-Cre-ZsGreen mice and ZsGreen mice. (B). Experimental timeline: visceral mechanical sensitivity was recorded from all animals before and after AAV injection, and then after VEGF_A_ instillations. The frequency-response curve of visceral mechanical sensitivity was recorded at the baseline, before AAV injection and instillations. No significant sex difference was detected. (C-D). No sex differences were detected in the withdrawal frequencies (C) or the response threshold (D) from naïve mice. (E). No significant difference was detected in the response threshold following CNO-injections when compared to the recordings from the same animals following saline injections. (F). Von Frey test performed four weeks after AAV injections. CNO injections significantly lowered the withdrawal frequencies and increased the response threshold in Gi-DREADD vector-injected TRPV1-Cre-ZsGreen mice but not Gi-DREADD vector-injected ZsGreen mice. (G). Von Frey test performed after intravesical instillations of VEGF_A_, at six weeks after AAV injections. CNO significantly lowered the withdrawal frequencies and increased the response threshold in Gi-DREADD vector-injected TRPV1-Cre-ZsGreen mice but not in Gi-DREADD vector-injected ZsGreen mice.

Next, a different AAV/mouse line combination was employed to further examine the visceral analgesic effect of pharmacogenetic inhibition in lumbosacral sensory neurons. AAV8-hSyn-DIO-Gi-DREADD-mCherry was injected *i*.*t*. into TRPV1-Cre-ZsGreen mice (Gi-DREADD group, N = 16) and age-matched ZsGreen mice lacking Cre recombinase (Control group, N = 7) (Fig 7A). Von Frey tests were used to probe the effects of AAV injections and sensory neuronal expression of Gi-DREADD on baseline visceral mechanical sensitivity (Fig 7B). Withdrawal frequencies and response threshold to visceral mechanical stimulation were compared in the same animals before and after AAV injections (Fig 7E–7G). Potential sex differences were assessed at the baseline (Fig 7C and 7D). No sex differences were identified in baseline visceral mechanical sensitivity (Fig 7C, p = 0.1923; Fig 7D, p>0.9999. Males: N = 15; Females, N = 8).

The potential “off-target” effects of CNO on visceral mechanical sensitivity were examined on all animals (Fig 7E). No significant differences were detected in the withdrawal frequencies (upper panel; CNO vs. saline, p = 0.2262) nor the response threshold (lower panel; p = 0.6487) following saline- or CNO administrations, further confirming our finding that CNO did not alter visceral mechanical sensitivity in the absence of Gi-DREADD. Four weeks after AAV injections, CNO administration significantly decreased the withdrawal frequencies to Von Frey filaments in TRPV1-Cre-Zsgreen mice when compared to the same animal following saline injections (Fig 7F; CNO vs. saline, p = 0.0055). CNO administration in TRPV1-Cre-ZsGreen mice also significantly decreased the response threshold when compared to the same animals after saline administration (Fig 7F, lower panel, p = 0.0229, paired *t-*test). Two-way ANOVA analysis showed significant differences between TRPV1-Cre-ZsGreen mice and ZsGreen mice (p = 0.0418). However, no statistical significance was detected when the response thresholds were directly compared between TRPV1-Cre-ZsGreen animals and ZsGreen animals after CNO administration.

All mice were then subjected to intravesical instillations of VEGF_A_. Von Frey test was performed on day 15 after the first instillation on all mice. As shown in Fig 7G upper panel, CNO administration significantly lowered the withdrawal frequencies in TRPV1-Cre-ZsGreen mice when compared to the same animals after saline administration (p<0.0001) or when compared to ZsGreen mice that also received CNO (p = 0.0007). In addition, CNO administration significantly increased the response threshold in TRPV1-Cre-ZsGreen mice when compared to ZsGreen mice (Fig 7G, lower panel, p = 0.0099) or the same animals after saline administration (p = 0.0009). Two-way ANOVA analysis revealed significant differences in both genotype (0.0021, TRPV-1-Cre-ZsGreen mice vs. ZsGreen mice) as well as drug treatment groups (0.0009, CNO vs. saline). Taken together, our data strongly suggest that decreasing peripheral afferent excitability with pharmacogenetic approaches leads to alleviation of the symptoms of bladder overactivity and visceral hypersensitivity.

## Discussion

In this study, we demonstrated that 1) intravesical instillations of VEGF_A_ did not affect the baseline nor maximum detrusor muscle contractility tested *in vitro*; nor did they change spontaneous voiding patterns *in vivo*, suggesting that VEGF_A_ exposure mostly targeted bladder innervation with minimal impact on the detrusor muscle itself; 2) bladder VEGF_A_ instillations enhanced nerve-mediated RTX-sensitive detrusor contractions, suggesting a predominant contribution of sensory neural pathways to neurogenic contractions; 3) activation of VEGF_A_ signaling in the urinary bladder increased gene and protein expression of TRPA1 channel in lumbosacral DRG, and induced visceral mechanical hypersensitivity; 4) silencing the activity of lumbosacral sensory neurons with Gi-DREADDs abolished the VEGF_A_-induced visceral mechanical hypersensitivity, suggesting a potential therapeutic strategy for alleviating visceral pain. Taken together, our data suggest that VEGF signaling in the urinary bladder differentially impacts pelvic sensitivity and voiding function in this mouse model of UCPPS, while predominantly affecting sensory nerve-mediated bladder contractions and pelvic mechanical sensitivity. In addition, we identified several sex differences at the behavioral level (pelvic mechanosensation), tissue level (baseline and nerve-evoked detrusor muscle contractility), and molecular level (VEGFR1 expression in lumbosacral DRG) that were independent from VEGF_A_ instillations.

### Bladder VEGF signaling in interstitial cystitis/painful bladder syndrome (IC/BPS)

VEGF signaling has been implied in the pathogenesis of IC/BPS [31]. VEGF and its receptors are expressed in urothelial, *lamina propria* and proposed intramural ganglionic cells within the bladder wall and upregulated in inflamed bladder [9, 32, 33]. Increased bladder VEGF expression has been linked to bladder pain severity in IC patients with glomerulations [9, 10, 34, 35]. In animal models, intravesical VEGF_A_ increased primary sensory neuronal excitability [16] and afferent nerve density in the mouse bladder [16, 36]. These findings suggested that bladder VEGF signaling may play a direct role in the pathogenesis of pelvic pain and urinary urgency observed in IC/BPS patients.

VEGF was also shown to be down-regulated following neovascularization, after the target organ starts to receive a sufficient supply of oxygen [37, 38]. Patients with IC exhibit a decreased blood flow in the bladders during the filling phase [39] and increased expression of hypoxia-inducible factor-1α (HIF-1) [10], which could upregulate VEGF as one of the mechanisms underlying hypoxia-induced angiogenesis [40]. However, the direct link between neovascularization and pain sensitivity has yet to be confirmed.

### TRPA1, but not TRPV1 is likely the key nociceptor underlying VEGFA-induced visceral hypersensitivity

In our study, we observed significant increases in gene and protein expression of TRPA1 channel in lumbosacral DRG neurons following VEGF_A_ instillations. TRPA1 is a member of the transient receptor potential (TRP) family of ion channels expressed in DRG neurons [41]. TRPA1-null mice displayed impaired responses to mustard oil, cold and mechanical stimuli, suggesting that TRPA1 contributes to the transduction of mechanical, cold, and chemical stimuli in nociceptor sensory neurons *in vivo* [41]. TRPA1 up-regulation was reported in bladder afferents in cyclophosphamide (CYP)-induced cystitis mice [42], and in L6-S1 DRG of CYP-induced cystitis rats [43]. In addition, TRPA1 antagonism reversed bladder hyperalgesia in mice with cystitis [42], as well as improved micturition and reversed overactive bladder-like symptoms in cystitis rats [43]. These reports strongly suggest that TRPA1 up-regulation plays an important role in bladder inflammation-induced visceral hypersensitivity and LUTS.

In addition to inflammation-induced models of LUTS, TRPA1 was also found to be upregulated in L6-S1 DRG in spinal cord injury (SCI) rats that exhibiting overactive bladder-like symptoms [44]. Administration of either TRPA1 antagonist or antisense oligodeoxynucleotide reversed LUTS and suppressed bladder afferent nerve hyperexcitability in SCI rats [44]. It is worth mentioning that TRPA1 was found to be upregulated in L6-S1 DRG only, but not in the corresponding segments of the spinal cord [44], suggesting that TRPA1 upregulation is important in regulating peripheral sensitivity in DRG neurons.

To explore the molecular mechanisms underlying VEGF_A_-induced visceral mechanical hypersensitivity, we examined the expression level of TRPA1 in the lumbosacral DRG from mice that received either VEGF_A_ or saline instillations. TRPA1 mRNA levels were significantly higher in lumbosacral DRG isolated from mice that received VEGF_A_ instillations compared to the saline group. Immunoreactivity of TRPA1 protein in lumbosacral DRG was also increased in VEGF_A_-instilled animals; this finding further confirmed that VEGF_A_-exposure to the urinary bladder upregulated TRPA1 channel in sensory neurons. However, one of the limitations of the study is that we did not distinguish bladder innervating DRG neurons from sensory neurons innervating other visceral organs. Future experiments with neuronal tracing will be useful to test if TRPA1 upregulation occurs in bladder sensory neurons in VEGF_A_-induced UCPPS model.

In addition to DRG neurons, we also detected TRPA1 immunoreactivity in lumbosacral DRG glia (S2 and S3 Figs). TRPA1 expression in satellite glial cells has been a subject of debate, with strong evidence showing both negative [45] and positive [46] expression of TRPA1 in DRG glia. A recent study reported that agonist-evoked, TRPA1-mediated Ca^2+^ elevations were enhanced in inflammation- or spare nerve injury-induced animal models of neuropathic pain [46], suggesting that satellite glial TRPA1 channel might contribute to primary sensitization. TRPA1 has also been shown to regulate resting calcium [47], Gamma-aminobutyric acid (GABA) transporter activity and inhibitory synapse efficacy [48] in astrocytes, the GFAP-positive glia in the central nervous system. Future experiments should be conducted to evaluate the potential role of satellite glial TRPA1 channel in bladder physiology and in VEGF_A_-induced visceral hypersensitivity.

Previous studies reported that VEGF signaling increases the expression of TRPA1 as well as TRPV1 channels in afferent terminals innervating tumor cells [15]. Furthermore, the same study showed that VEGFR1 activation facilitates the transport and insertion of TRPV1 into the plasma membrane in primary sensory neurons [15]. It has also been reported that TRPA1 and TRPV1 channels can be co-transported to the plasma membrane in sensory neurons following neuroinflammation [49]. Therefore, we examined the expression levels of TRPV1 channels in the same animals with increased expression of TRPA1 in sensory ganglia. We found no evidence of VEGF_A_-induced changes in TRPV1 expression at both mRNA and protein levels. Our results are consistent with the previous study in CYP-induced cystitis mice [42]. Together, our data suggest that in the context of visceral afferent sensitization associated with VEGF signaling, TRPV1 and TRPA1 channels seem to be differentially regulated.

### VEGFR1 expression in bladder afferent neurons: Can sex difference explain susceptibility?

One interesting finding in our study is the sex differences of VEGFR1 expression in lumbosacral DRG. Lumbosacral DRG isolated from female mice consistently showed higher VEGFR1 immunoreactivity, which could be linked with higher sensitivity to urinary VEGF in females. One of the limitations of our approach was that we did not track the changes in females at different phases of the estrous cycle due to large numbers of both males and females included in the study. However, we acknowledge that thresholds of pelvic sensitivity in females do change during the estrous cycle [50], and should be tracked in female exclusive studies. No significant differences were found in the mRNA expression nor in the immunoreactivity of VEGFR1 and R2 in lumbosacral DRG between VEGF_A_-instilled and saline-instilled mice of both sexes, suggesting that repeated VEGF_A_ exposure over two weeks was not sufficient for inducing changes in VEGFR1 and VEGFR2.

We have attempted to compare the expression levels of VEGFR1 and VEGFR2 in primary afferents in the bladder wall between saline- or VEGF_A_-instilled mice. Unfortunately, commercially available antibodies against VEGFR1 and VEGFR2 failed to produce consistent labeling in the urinary bladder. Therefore, only lumbosacral DRG expression levels of VEGFR1 and VEGFR2 were tested.

### The role of nNOS in visceral pain/mechanical hypersensitivity

In this study, we have observed a significantly lower nNOS mRNA expression in lumbosacral DRG following intravesical instillations of VEGF_A_. The expression of nNOS has been identified in subsets of DRG neurons [51, 52] as well as nerve afferents in the bladder wall [53, 54] in different experimental animal models [55]. Bladder VEGF signaling increases substance P immunoreactivity in sensory nerve fibers in the bladder [36]. It is also known that nNOS mRNA is negatively regulated by substance P in DRG and spinal cord of the rats [56]. In addition to the published results, our data suggest that nNOS production has been downregulated in primary sensory neurons after repeated VEGF_A_ exposure to the bladder lumen and could be a downstream effect from increased substance P expression in sensory fibers innervating the urinary bladder.

The changes in nNOS expression have been reported in animal models of mechanical hypersensitivity. Several animal models of peripheral nerve injury [54, 57–59], including spinal cord injury have shown increases in nNOS expression shortly after the peripheral insult. Differential regulation of nNOS expression during disease progression were also reported between lumbar and sacral DRG [51, 52, 60]. These data suggested that nNOS expression is very dynamic and possibly regulated by peripheral nerve activity. Interestingly, many have reported unchanged spinal dorsal horn expression of nNOS while nNOS was significantly upregulated in the lumbosacral DRG following peripheral insults [58, 61, 62], suggesting that nNOS regulation in DRG neurons might act as an important inhibitory mechanism to prevent peripheral nociceptive sensitization from transmitted to the spinal neurons after an initial peripheral insult.

Most of the studies examining nNOS expression in the context of urinary dysfunction utilized animal models of bladder outlet obstruction. Significant increases in nNOS expression were often observed in DRG [60] and in the bladder wall [63–65] following bladder outlet obstruction. However, significant decreases in nNOS expression have also been reported [66, 67]. Giving the dynamic expression of nNOS, these data could result from different timing/disease severity of the PBOO model when the data were obtained. Importantly, the changes in nNOS expression were also reversed by removing PBOO [63] as well as by intravesical electrical stimulation in spinalized rats [54], suggesting the reversibility of nNOS regulation in primary sensory neurons and afferent terminals.

### Manipulation of sensory neuronal signaling to improve bladder sensitivity

Anti-VEGF treatment was previously shown to prevent afferent nerve plasticity [36], to decrease bladder pain [68], and to increase bladder capacity and voiding volume [69] in rodent models of CYP-induced cystitis. Recently, it was reported that VEGFR2 antagonism also elevated voiding volume in healthy control rats, suggesting a physiological role of VEGF_A_ signaling in bladder filling. However, given the various important physiological functions of VEGF, pharmacological disruption of VEGF signaling in patients would need to be restricted to the bladder to selectively target bladder afferent innervation. In this study, we tested the feasibility of using inhibition of sensory neuronal excitability as an alternative approach to antagonizing VEGF signaling. Our data demonstrated that sensory neural inhibition effectively reduced visceral sensitivity without significantly affecting physiological bladder function. Our data also suggested that non-invasive, remote control of peripheral neuronal activity can be achieved using AAV-mediated gene therapy similar to that reported in other organ and tissues [70].

One of the innovative aspects of our study was the control of excitability and activity of lumbosacral sensory neurons *in vivo* and remotely with DREADDs. DREADDs are a group of engineered GPCRs that couple with endogenous signaling pathways [30]. Since their development, DREADDs have been extensively used to manipulate the activity of neural [71–75] and non-neural [76, 77] cells *in vivo* to probe the roles of particular populations of cells in complex circuits. When the Gi-coupled DREADD, Gi-DREADD is activated in neurons, it engages Gi-GPCR signaling pathways, which subsequently activate potassium channels and cause cell hyperpolarization, thereby, decreasing its excitability. Therefore, Gi-DREADD has been commonly used to inhibit neuronal activity and decrease signal transduction in neural circuits [78]. Gi-DREADD can be expressed transgenically [21] or with the help of viral vectors [79]. In this study, we demonstrated high tropism of Gi-DREADD expression in TRPV1-positive neurons in lumbosacral DRG, and in the dorsal horn of the spinal cord using TRPV1-Cre mice and hSyn promotors in AAV vectors. Our approach enabled selective inhibition in lumbosacral sensory neurons, including the neurons that carry afferent signals from the urinary bladder. The Gi-DREADD agonist, CNO, did not induce any changes in control vector injections in any of the parameters we analyzed, enabling selective inhibition of visceral afferent signal in awake, free-moving animals.

One caveat in our approach was the broad neuronal inhibition among both bladder and non-bladder innervating neurons in the lumbosacral DRG. Engineering retrograde AAVs that are effective in transducing the majorities of the bladder innervating DRG neurons via bladder injection/instillations will be the next step to confirm the feasibility of this approach for selective manipulation of subset of DRG neurons innervating the urinary bladder.

By using AAV-mediated pharmacogenetic inhibition, we identified bladder afferent signaling as key contributor to VEGF_A_-induced visceral hypersensitivity. Our data suggests that pelvic pain symptoms in UCPPS patients could be potentially improved by decreasing peripheral afferent activity alone. Additional studies are needed to reveal the differential contribution of peripheral and central afferent pathways in VEGF_A_-induced visceral hypersensitivity necessary to identify additional therapeutic targets such as new sites for neuromodulation-based therapies in UCPPS patients. Long-term studies should also be planned to assess the potential for treating chronic UCPPS symptoms following AAV-mediated gene manipulation.

## Supporting information

S1 Fig Western blotting validation of rabbit anti-TRPA1 antibody from St John’s laboratory, STJ193121. In mouse tissue, STJ193121 exhibited a single band of the expected molecular weight (~120 kDa).
(TIF)

S2 Fig Anti-TRPA1 antibody validation using immunofluorescence labeling in mouse DRGs. (A). Three different anti-TRPA1 antibodies were used on frozen mouse DRG sections. ZsGreen signaling is not amplified in these images because the ZsGreen antibody and some TRPA1 antibodies are raised in the same host (rabbit). TRPA1 primary antibodies were amplified with Alexa 594 conjugated Donkey secondary antibodies from Invitrogen Molecular Probes. All TRPA1 antibodies were chosen at the highest concentration suggested by manufacture. (B) Overlay of TRPA-1 and ZsGreen expression in TRPV1-positive neurons in mouse lumbosacral DRG. Scale bar: 20 μm.
(TIF)

S3 Fig TRPV-1-driven and Cre-dependent ZsGreen expression in a sensory neurons and satellite glial cells in lumbosacral DRG. Antibody against brain lipid binding protein (BLBP), a glial marker, was used to label the cytosol of satellite glial cells in DRG. Scale bar: 20 μm.
(TIF)

S1 Data (XLSX)

S2 Data (XLSX)

S3 Data (XLSX)

S4 Data (XLSX)

S1 File (PDF)

## Abbreviations

BLBP: Brain lipid-binding protein
CNO: Clozapine-N-oxide
DREADDs: Designer Receptors Exclusively Activated by Designer Drugs
DRG: Dorsal root ganglia
GABA: Gamma-aminobutyric acid
GFAP: Glial fibrillary acidic protein
Gapdh: Glyceraldehyde 3-phosphate dehydrogenase
GPCR: G protein-coupled receptors
IC: Interstitial cystitis
MAPP: Multidisciplinary Approach to the Study of Chronic Pelvic Pain
nNOS: Neuronal Nitric Oxide Synthase
BPS: Painful bladder syndrome
PNS: Peripheral nervous system
RTX: Resiniferatoxin
hSyn: Human synapsin 1 gene promoter
TTX: Tetrodotoxin
TRPA1: Transient receptor potential cation channel subfamily A member 1
TRPV1: Transient receptor potential cation channel subfamily V member 1
TUBB3: Tubulin Beta 3 Class III
UCPPS: Urologic chronic pelvic pain syndrome
VEGF: Vascular endothelial growth factor
VEGFR1: VEGF receptor 1 and
VEGFR2: VEGF receptor 2
BSMSC: Bladder smooth muscle strip contractility recordings
