# Supplementary figures and images for: Pharmacogenetic inhibition of lumbosacral sensory neurons alleviates visceral hypersensitivity in a mouse model of chronic pelvic pain
Source: PLoS One. 2022 Jan 25;17(1):e0262769. doi: 10.1371/journal.pone.0262769 (PMC8789164; doi:10.1371/journal.pone.0262769)

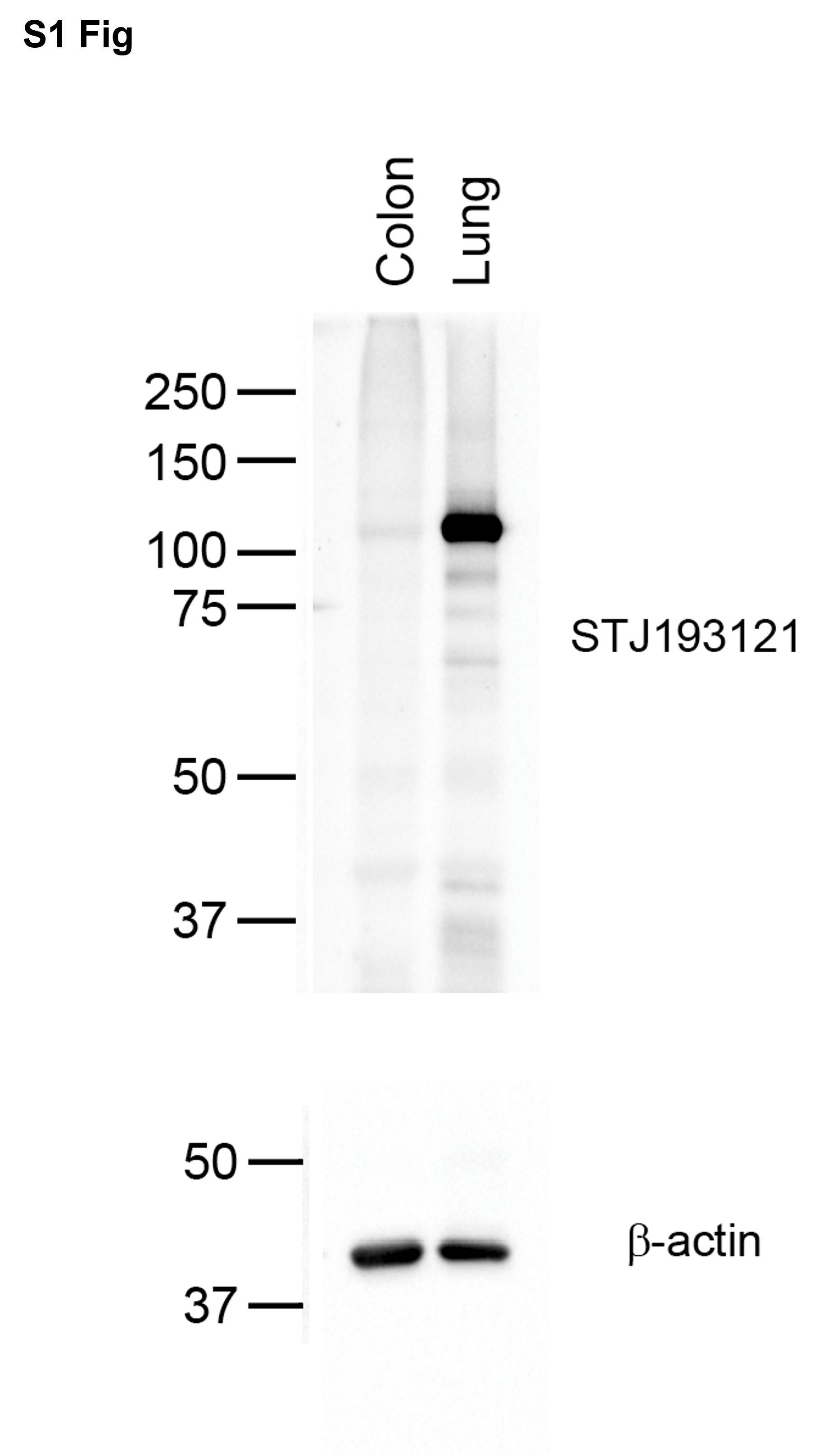

Supplement: S1 Fig — In mouse tissue, STJ193121 exhibited a single band of the expected molecular weight (~120 kDa). (TIF) [file pone.0262769.s001.tif]

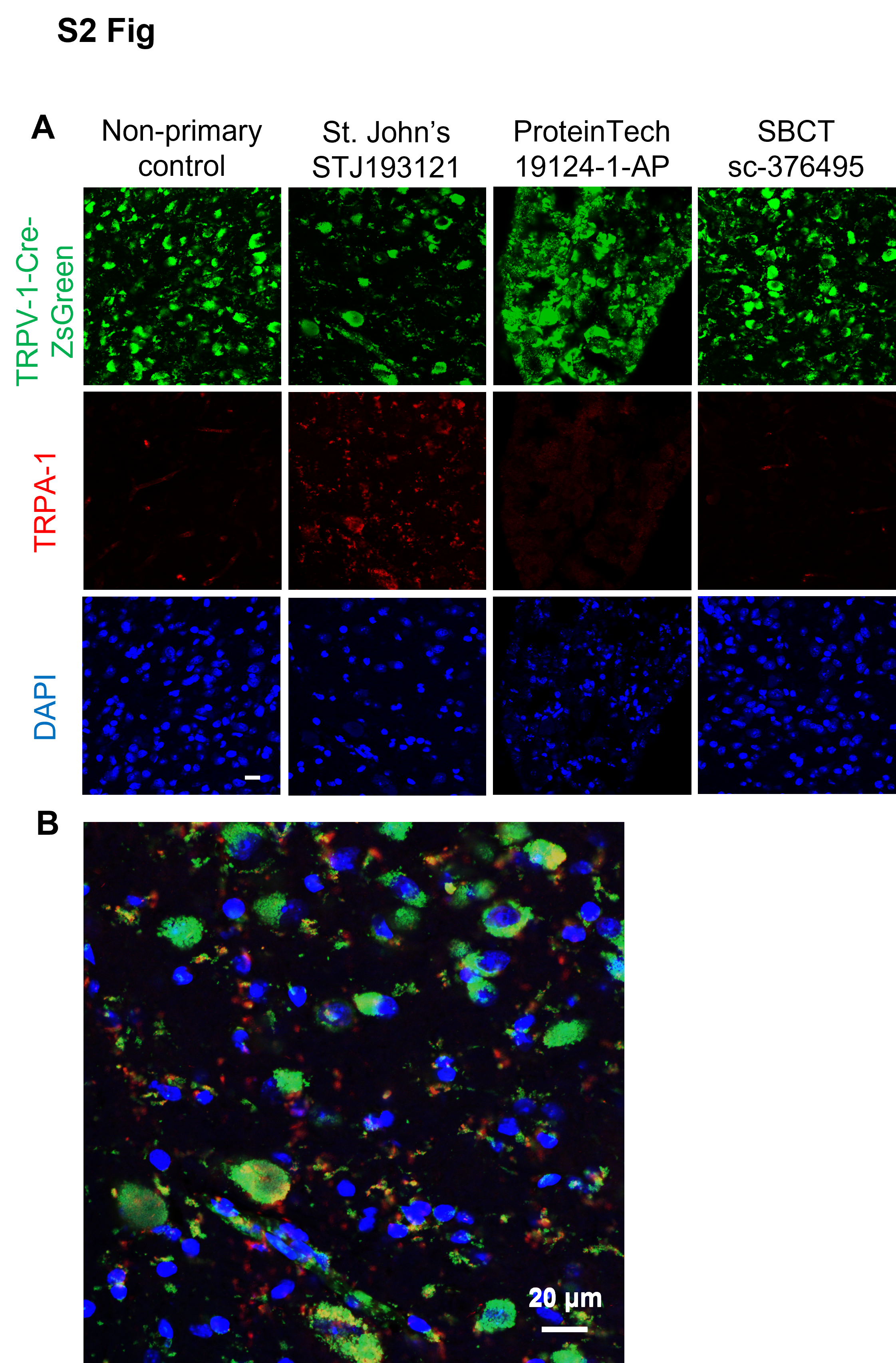

Supplement: S2 Fig — (A). Three different anti-TRPA1 antibodies were used on frozen mouse DRG sections. ZsGreen signaling is not amplified in these images because the ZsGreen antibody and some TRPA1 antibodies are raised in the same host (rabbit). TRPA1 primary antibodies were amplified with Alexa 594 conjugated Donkey secondary antibodies from Invitrogen Molecular Probes. All TRPA1 antibodies were chosen at the highest concentration suggested by manufacture. (B) Overlay of TRPA-1 and ZsGreen expression in TRPV1-positive neurons in mouse lumbosacral DRG. Scale bar: 20 μm. (TIF) [file pone.0262769.s002.tif]

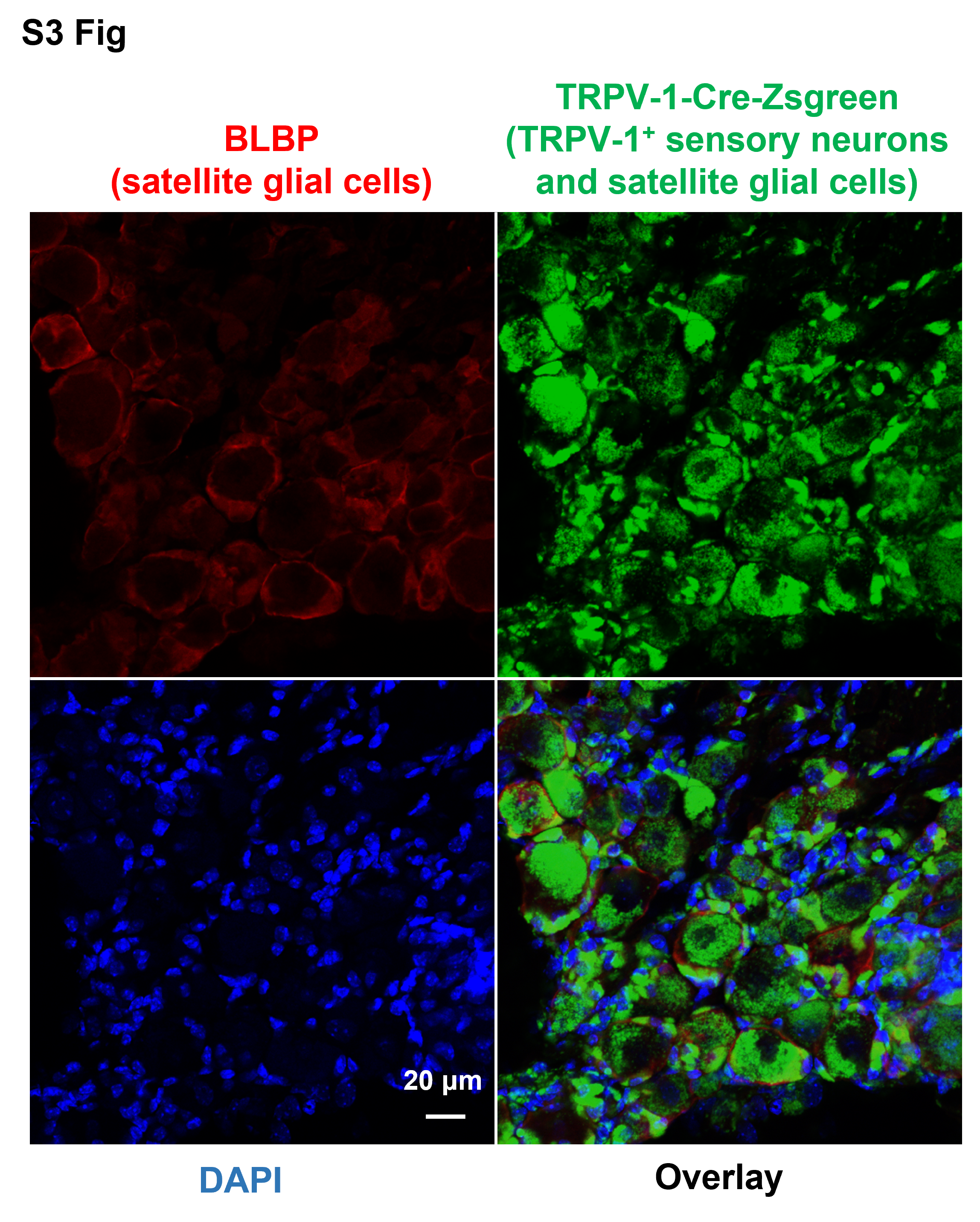

Supplement: S3 Fig — Antibody against brain lipid binding protein (BLBP), a glial marker, was used to label the cytosol of satellite glial cells in DRG. Scale bar: 20 μm. (TIF) [file pone.0262769.s003.tif]
