# Supplementary material for: Pharmacogenetic inhibition of lumbosacral sensory neurons alleviates visceral hypersensitivity in a mouse model of chronic pelvic pain
Source: PLoS One. 2022 Jan 25;17(1):e0262769. doi: 10.1371/journal.pone.0262769 (PMC8789164; doi:10.1371/journal.pone.0262769)
Supplement: S1 File — (PDF) [file pone.0262769.s008.pdf]

This presentation should have included all images analyzed in the paper plus some images (from CG/Chat-Cre-ZsGreen) mice for another project

Due to size limit, it is printed to pdf.

Rationale:
Previous report in cancer research indicated that VEGFR1 activation let to increased thermal and mechanical sensitivity via TRPV-1 and TRPA-1 mediated pathways, respectively.
Western blot analysis showed that VEGFR1, but not VEGFR2, was significantly upregulated in the lysates of ipsilateral L3–L4 lumbar DRGs isolated from tumor-bearing mice as compared with sham-treated animals.

Question: Does the expression level of VEGFRs, TRPV1 and TRPA1 change in DRG sensory neuronal somata in response to bladder $VEGF_A$ instillation?

Experimental design: Littermates receive bladder instillation of either $VEGF_A$ or saline. Immunostaining for VEGFR1, R2, TRPV-1, and TRPA1 in L5/L6-S2 DRG.

The 488 channel was reserved for ZsGreen signal, 594 channel was used for anti-VEGFR1, anti-VEGFR2, or anti-TRPV-1. DAPI was used to reveal the cell nuclei.

Rosa-ZsGreen +/+ mice (no naïve ZsGreen) | Non-primary | DAPI | Overlay

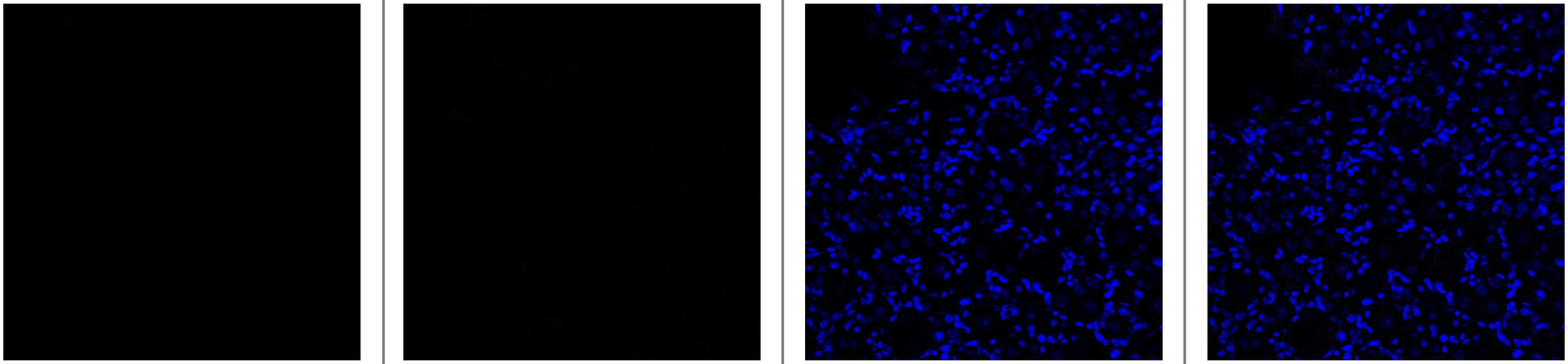

Little autofluorescence, used the same setting to document antibody staining

Antibody against VEGFR1 exhibits broad staining in DRG neurons and potentially satellite glia cells (1), with strong perinuclear staining (2). The VEGFR1 signal is also detected in the cytosol of small diameter neurons (3).

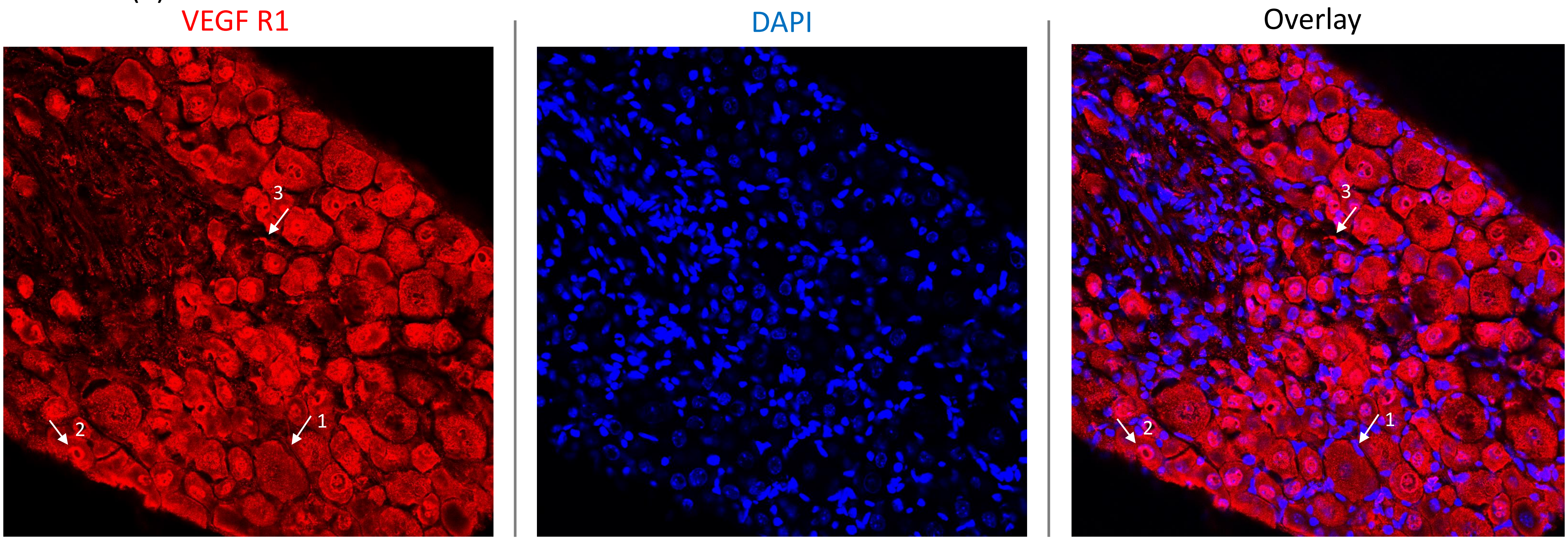

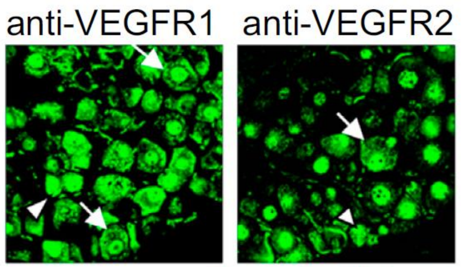

20190328 RGs/PBSVR1
RG female_R0165_40x_1
Saline Instillation

Image analysis strategy

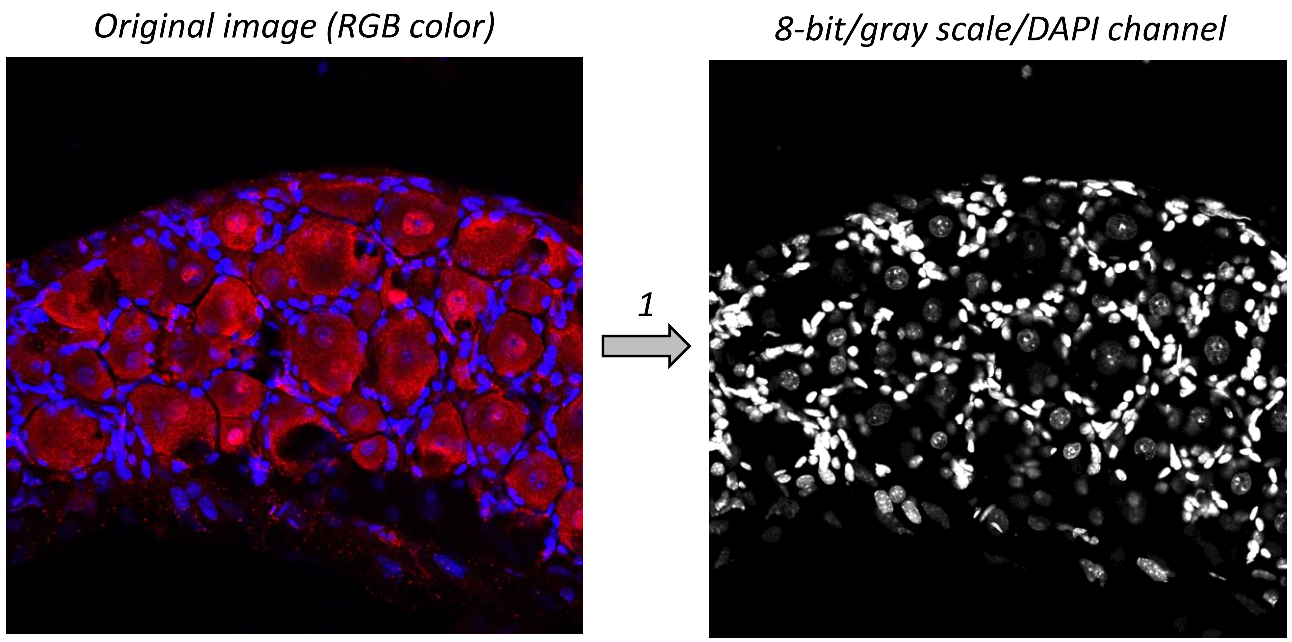

Pros: non-biased analysis of the signal intensity of VEGF R1 in the whole DRG

Cons: no differentiation between expression in different cell types (neuronal vs glia) or subcellular locations (cytosol vs nuclei)

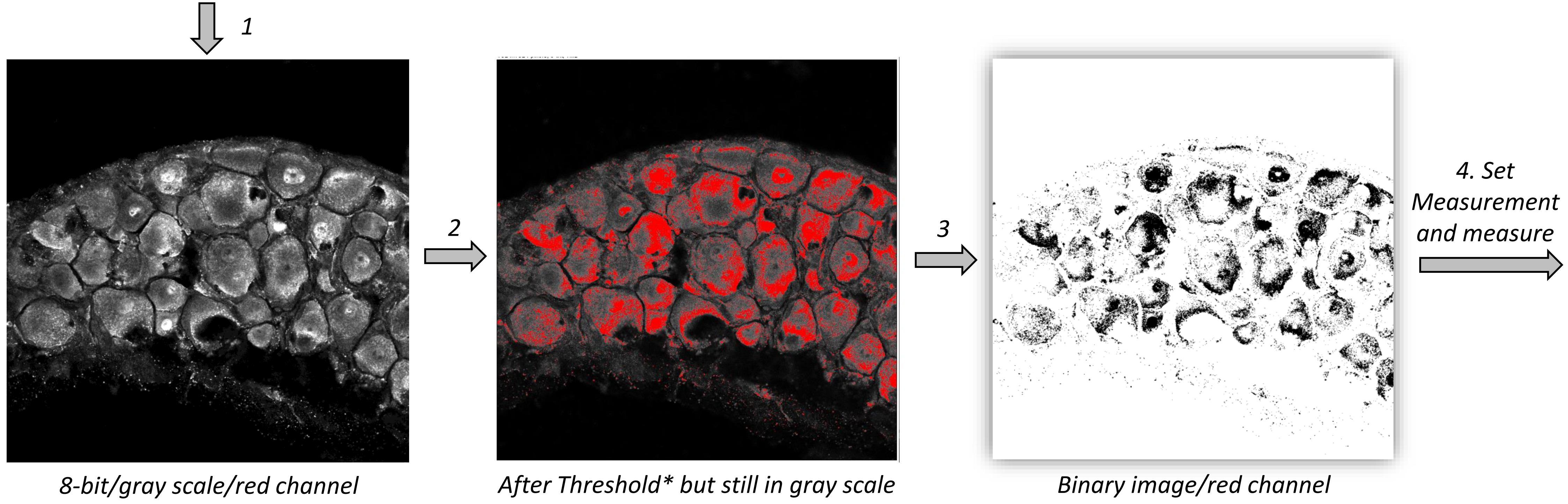

*Threshold is set based on non-primary images from the same experiments (day - should have been animals)

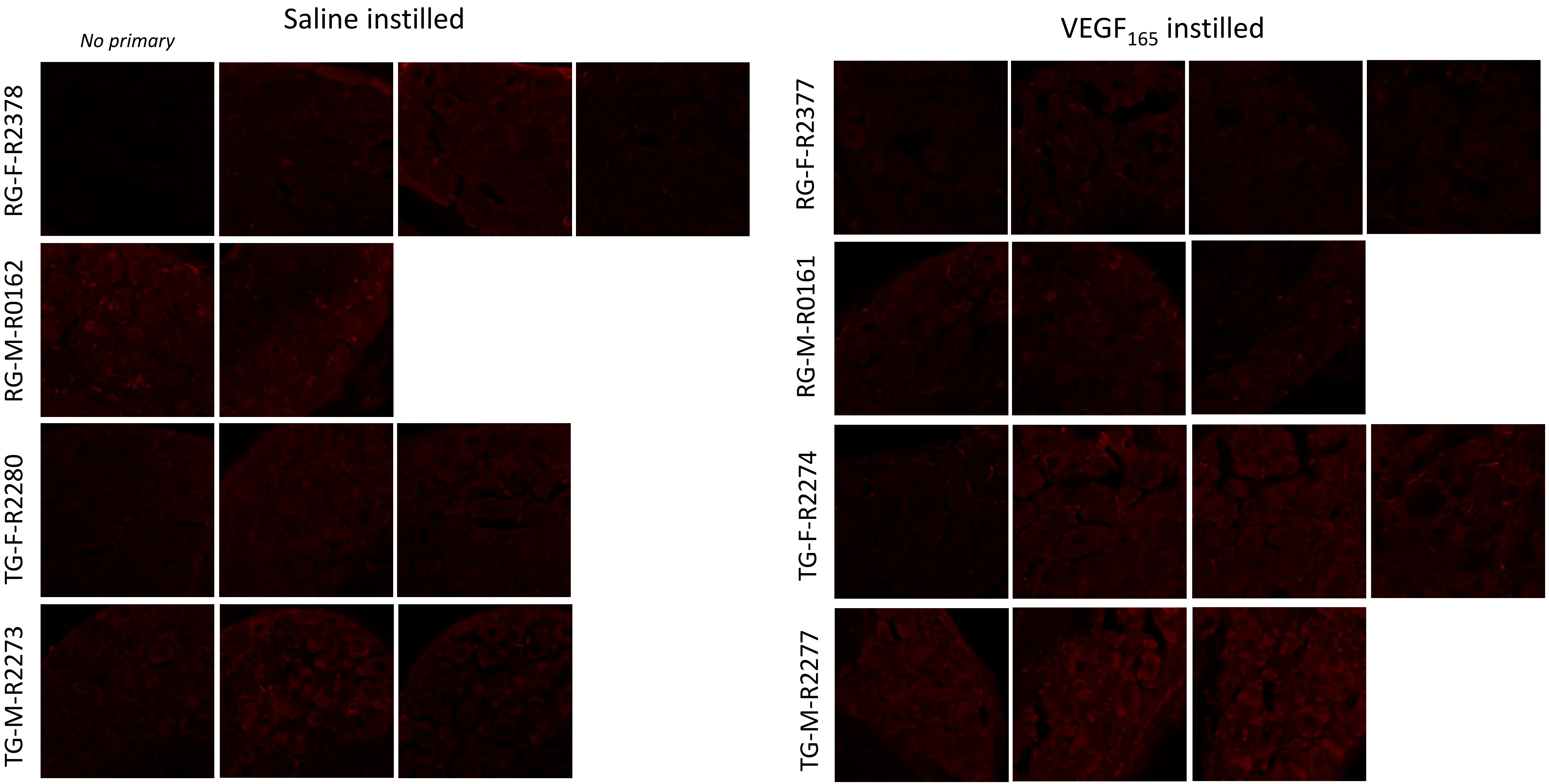

VEGF R1
Staining day 1

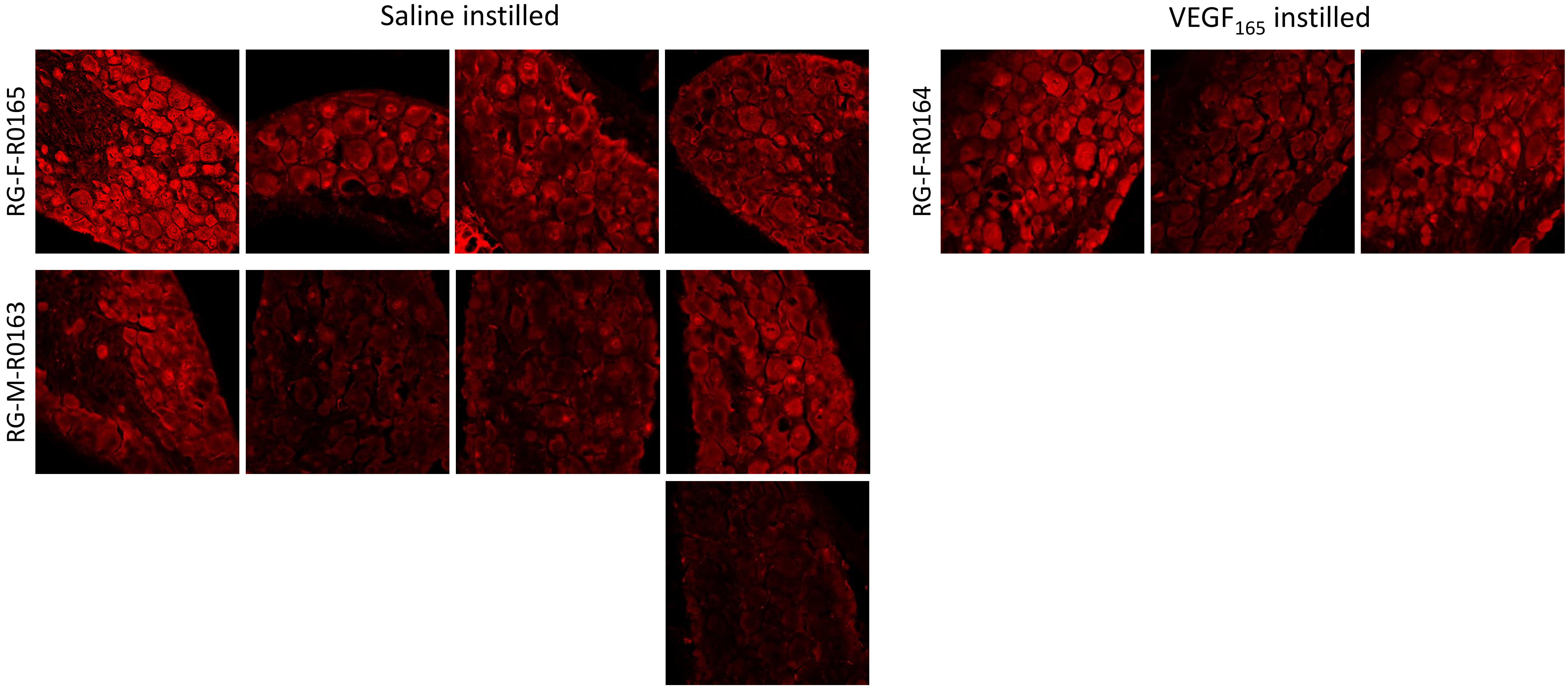

VEGF R1
Staining day 2

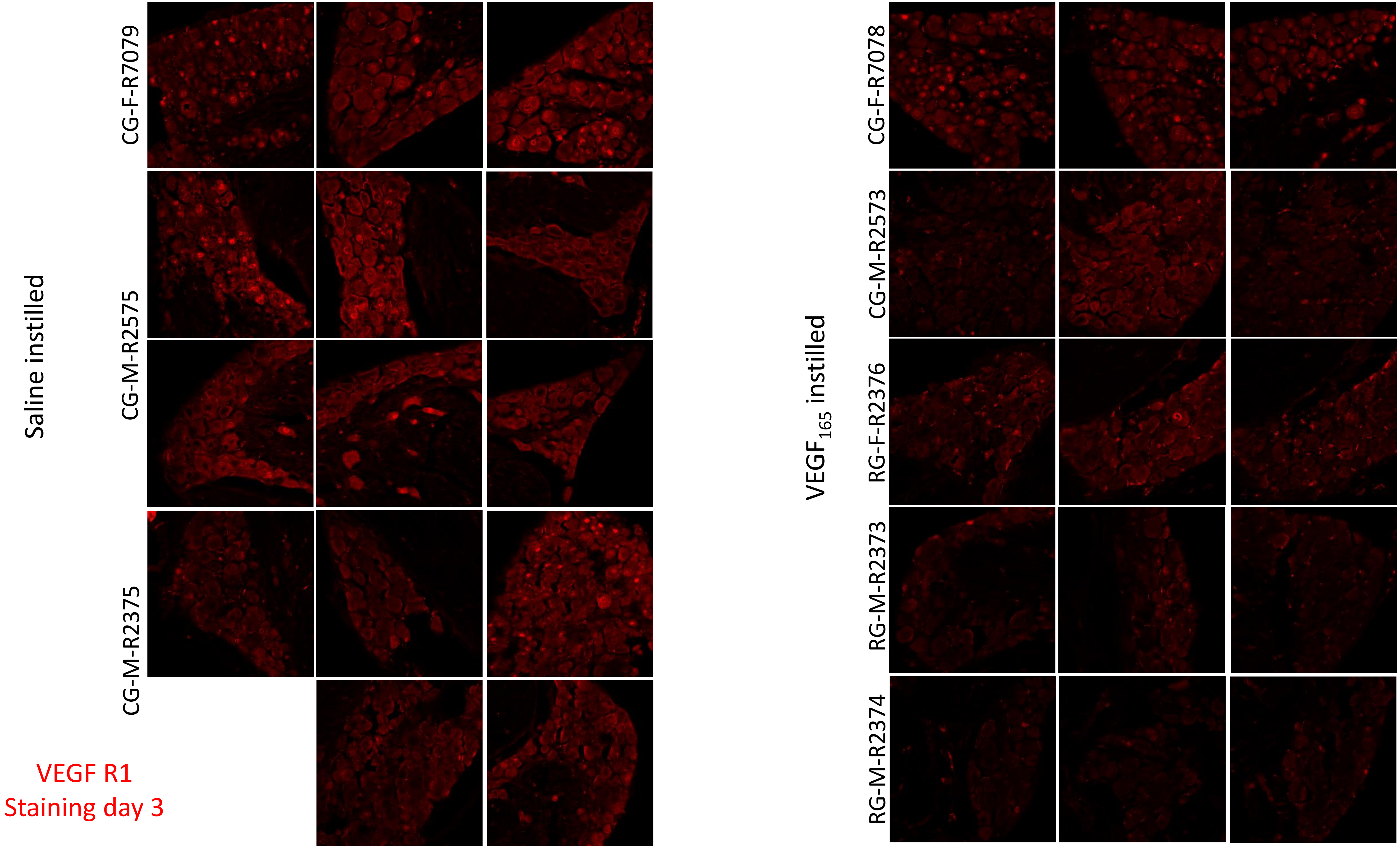
Saline instilled
CG-F-R7079
CG-M-R2575
CG-M-R2375
VEGF165 instilled
CG-F-R7078
CG-M-R2573
RG-F-R2376
RG-M-R2373
RG-M-R2374
VEGF R1
Staining day 3

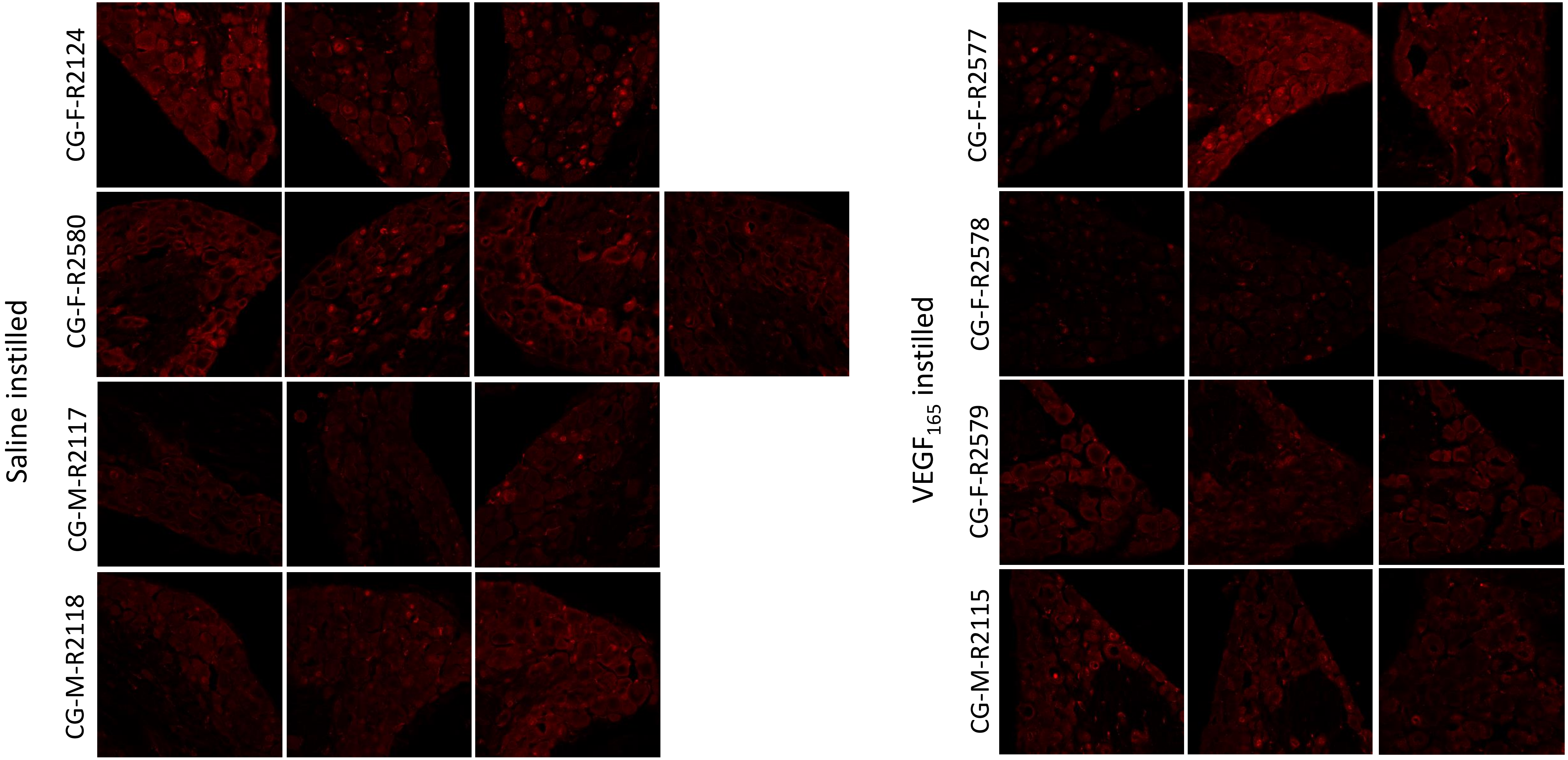

VEGF R1
Staining day 4 CGs

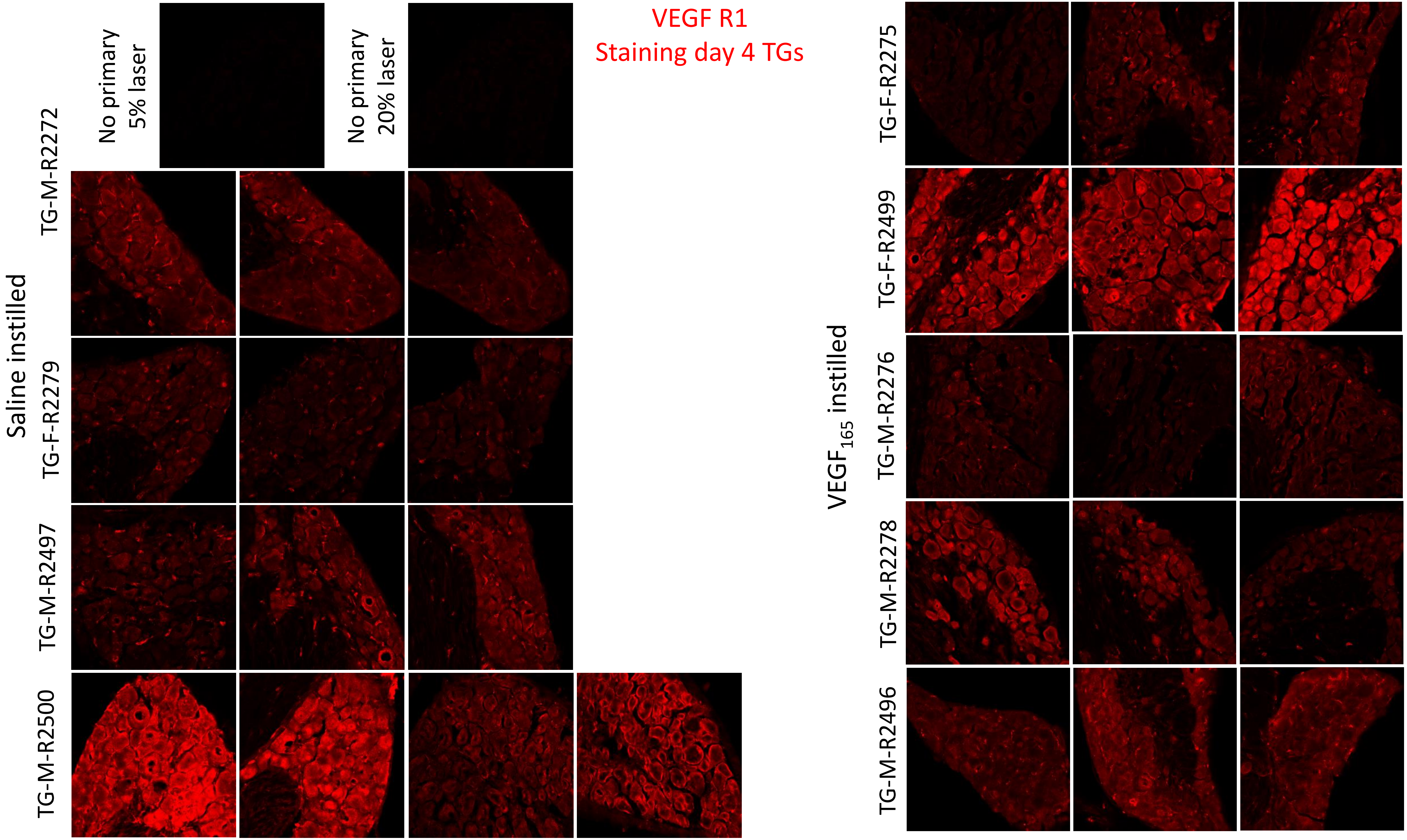
VEGF R1
Staining day 4 TGs
No primary 5% laser
No primary 20% laser
Saline instilled
TG-M-R2272
TG-F-R2279
TG-M-R2497
TG-M-R2500
$VEGF_{165}$ instilled
TG-F-R2275
TG-F-R2499
TG-M-R2276
TG-M-R2278
TG-M-R2496

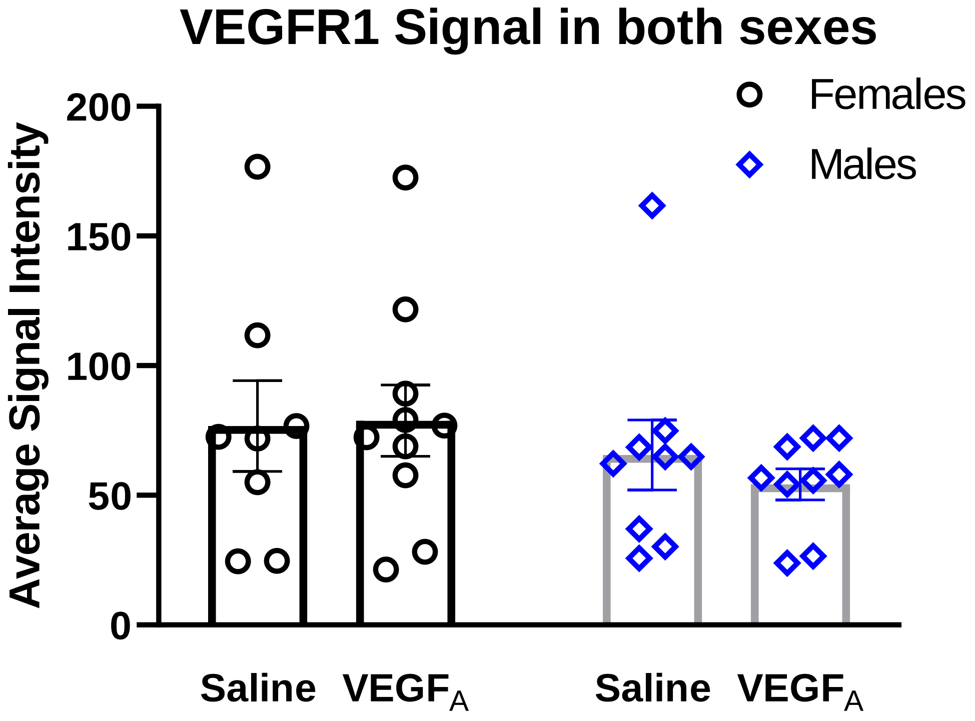

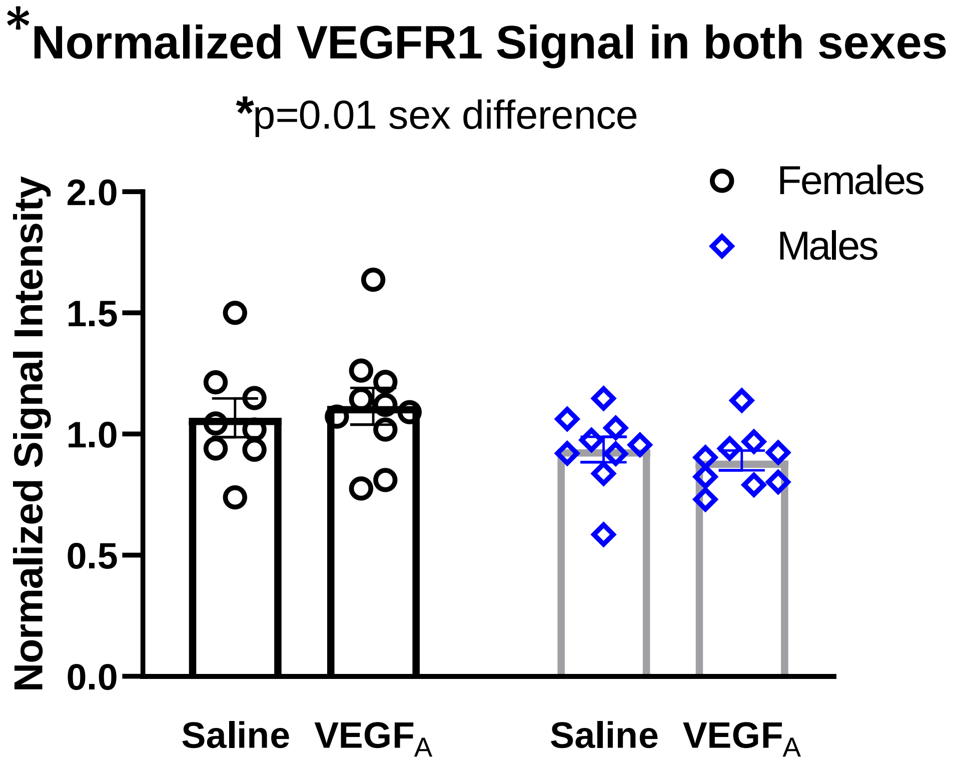

Total count	36	M	F
Saline	17	9	8
VEGF165	19	9	10

** Normalized to the average intensity of the saline-instilled tissue from the same staining day.*

Two schools of thoughts:

VEGFR-2 is robustly expressed by endothelial cells throughout the extensive DRG capillary network, but not found in sensory neurons or other nonendothelial cells of the DRG.

A

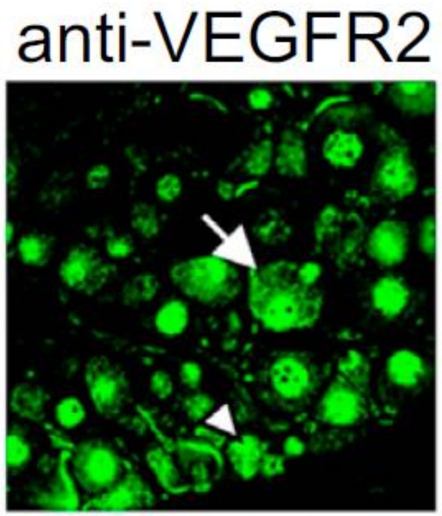

B

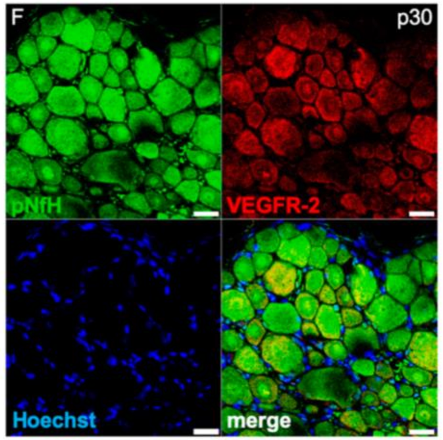

C

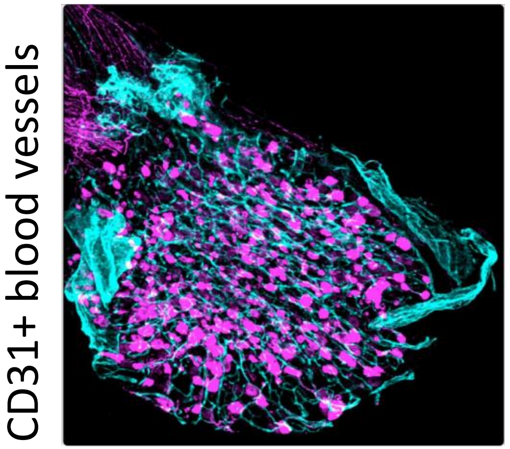

D

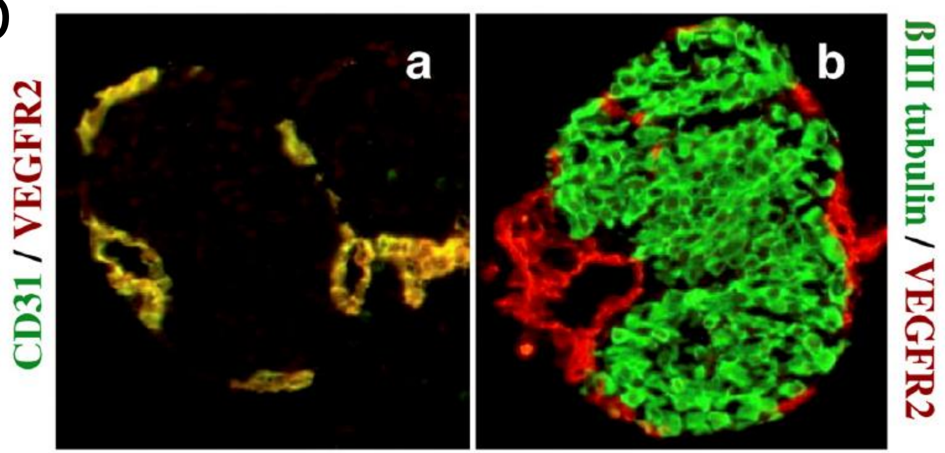

D Freshly dissected

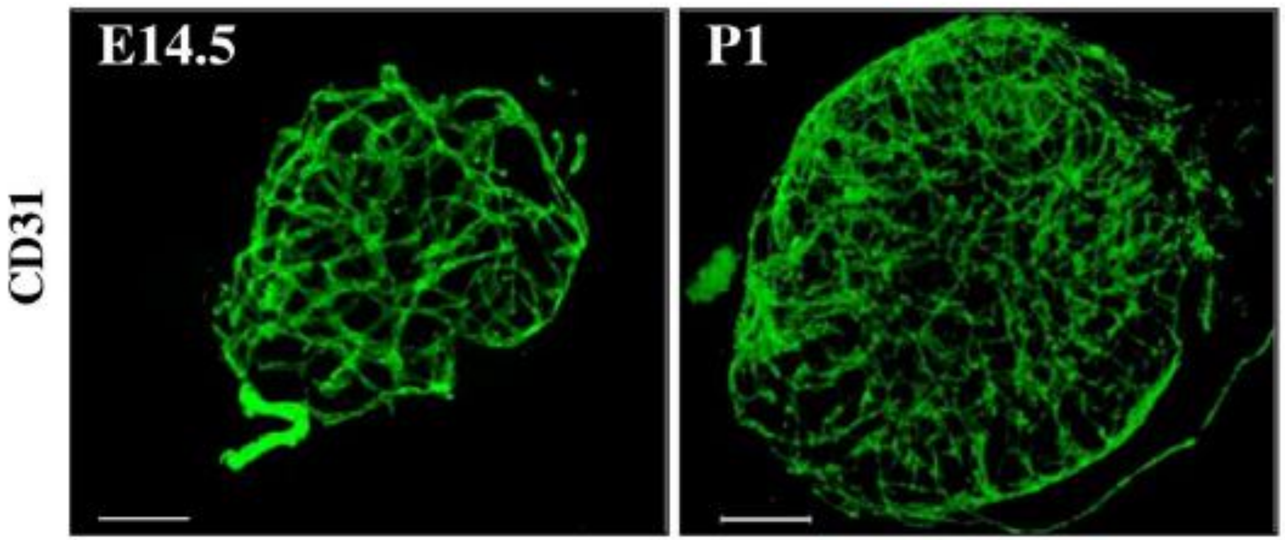

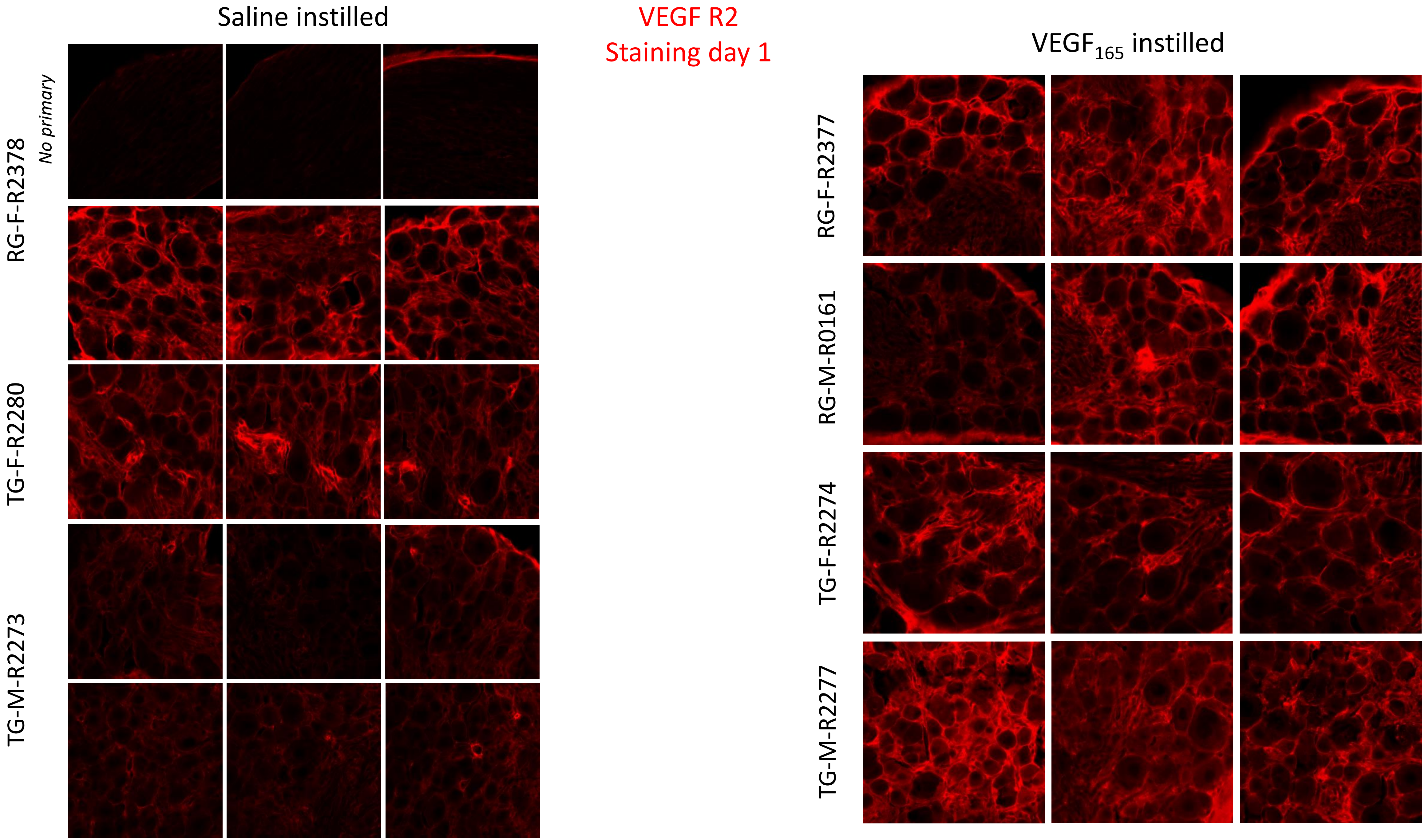
VEGF R2
Staining day 1
Saline instilled
No primary
RG-F-R2378
TG-F-R2280
TG-M-R2273
VEGF165 instilled
RG-F-R2377
RG-M-R0161
TG-F-R2274
TG-M-R2277

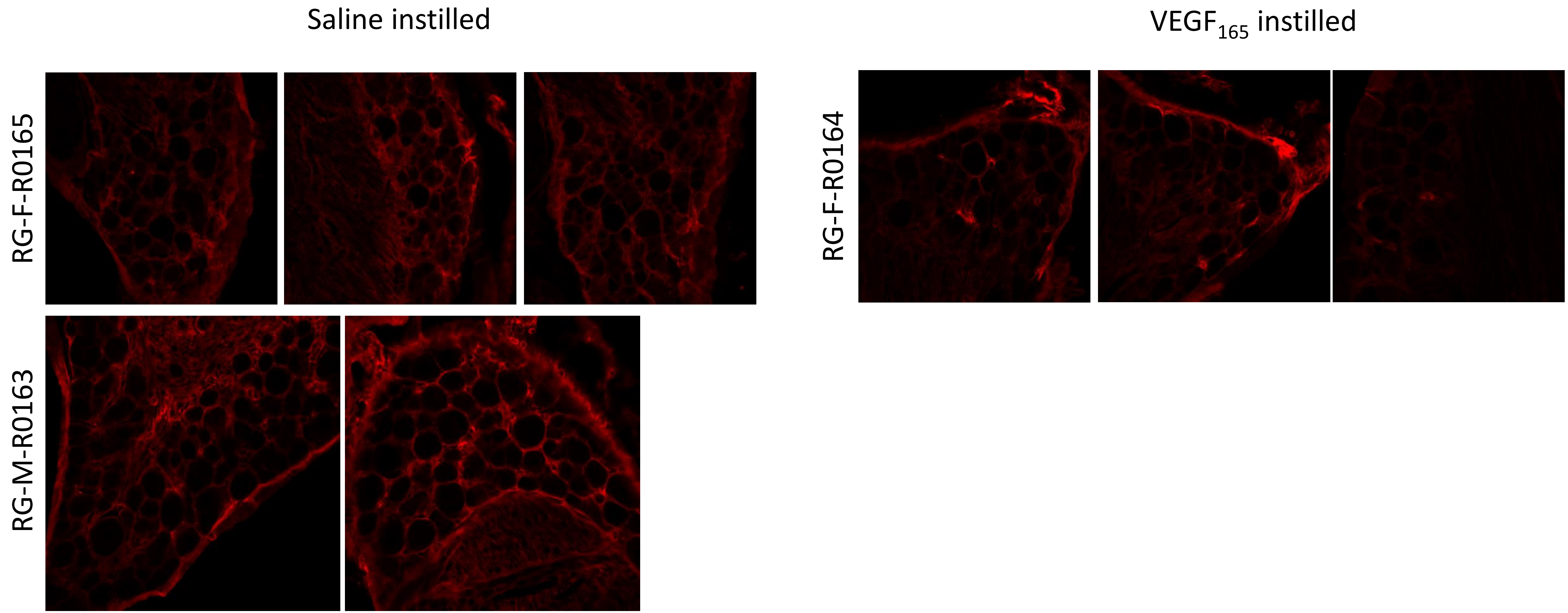

VEGF R2
Staining day 2

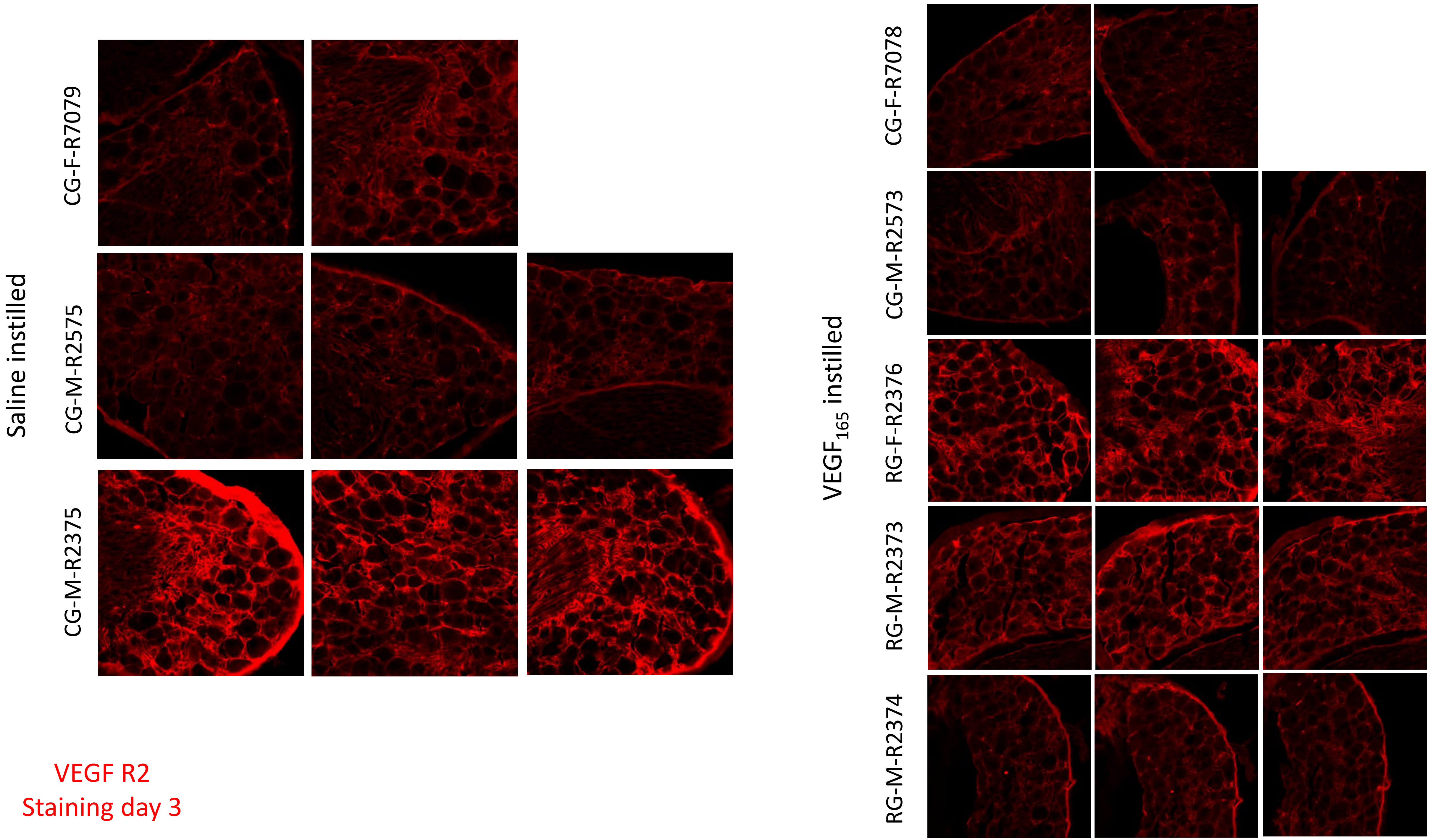

Saline instilled
CG-F-R7079
CG-M-R2575
CG-M-R2375
VEGF165 instilled
CG-F-R7078
CG-M-R2573
RG-F-R2376
RG-M-R2373
RG-M-R2374
VEGF R2
Staining day 3

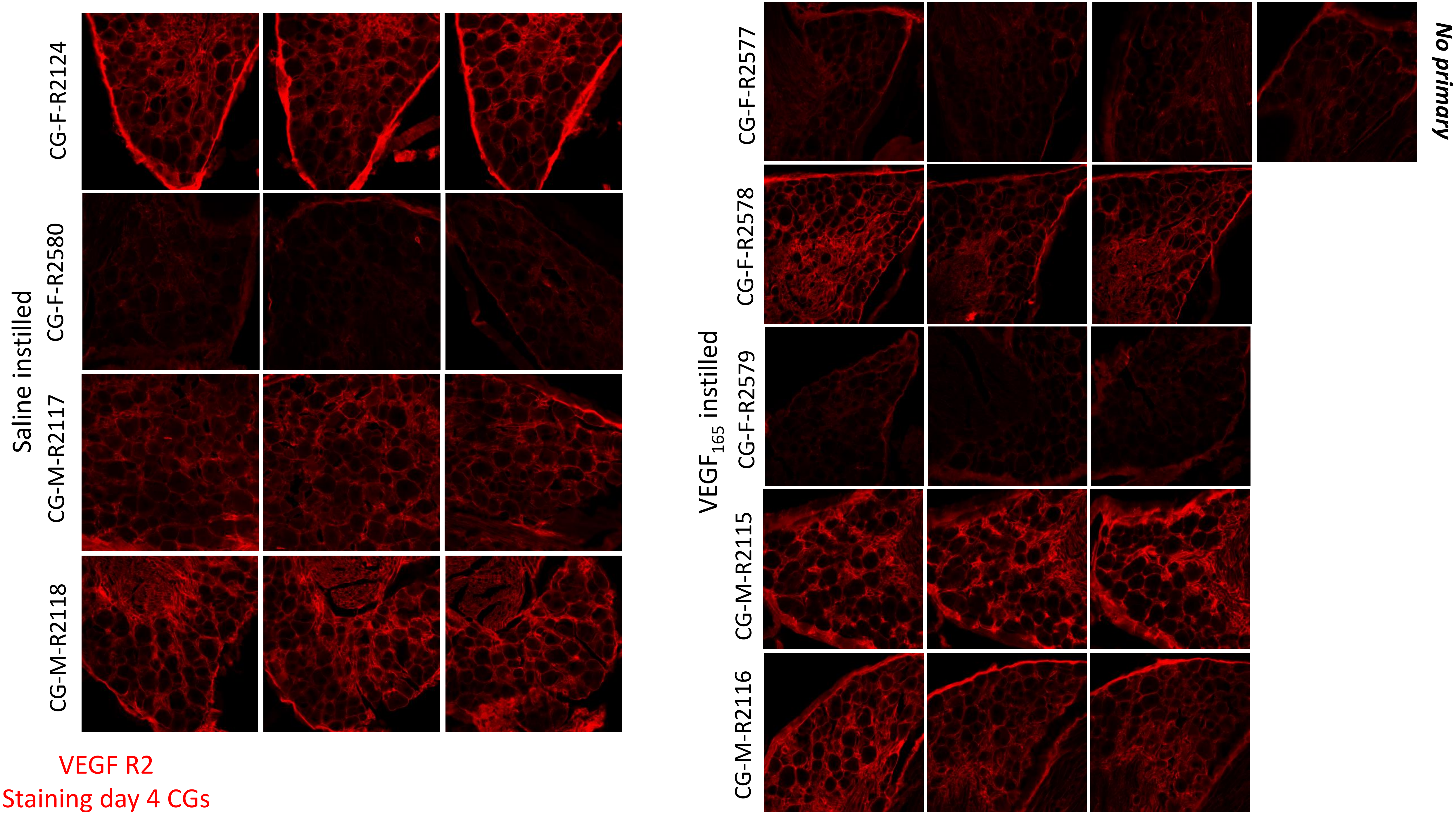

VEGF R2
Staining day 4 CGs

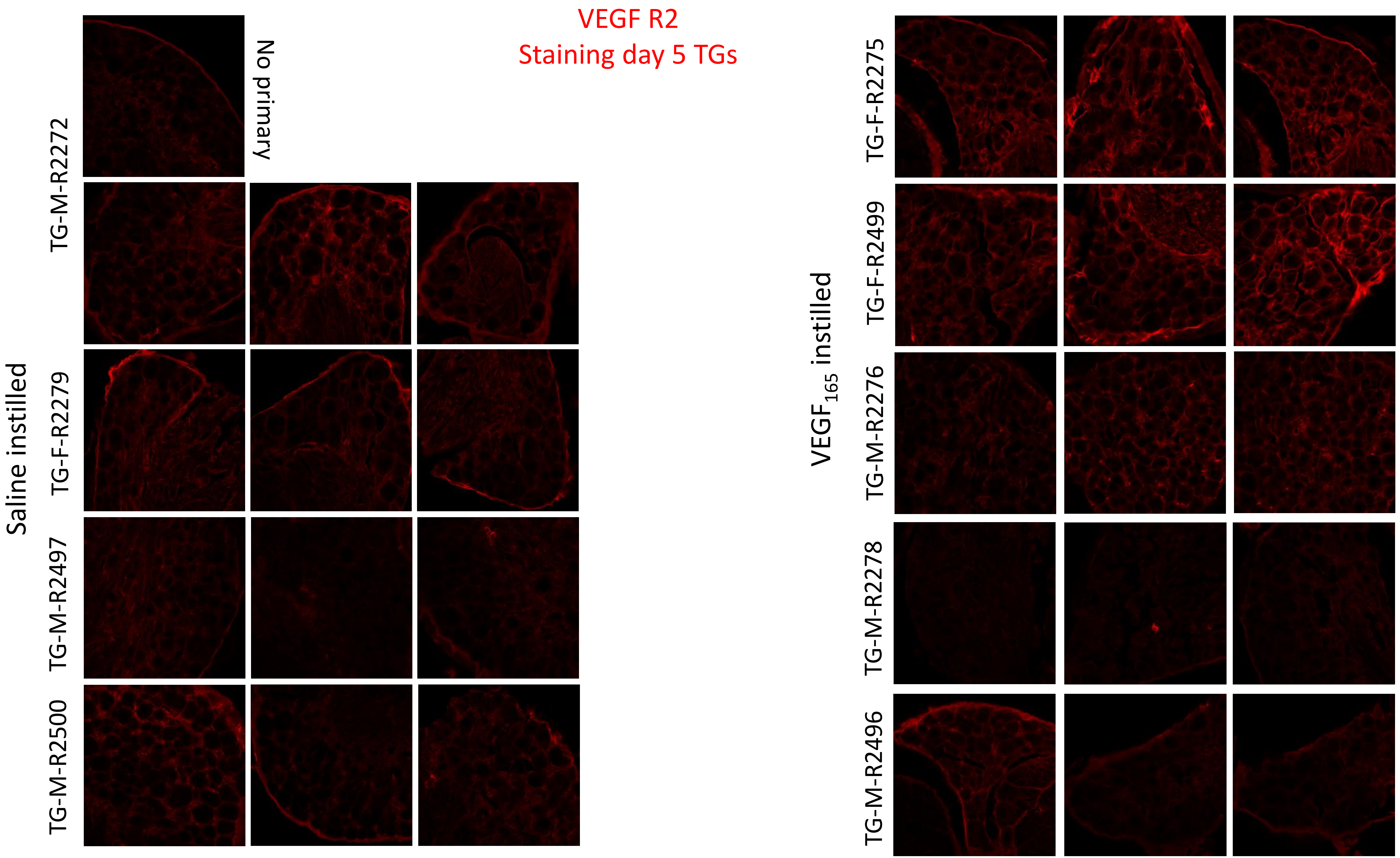
VEGF R2
Staining day 5 TGs
No primary
Saline instilled
TG-M-R2272
TG-F-R2279
TG-M-R2497
TG-M-R2500
VEGF165 instilled
TG-F-R2275
TG-F-R2499
TG-M-R2276
TG-M-R2278
TG-M-R2496

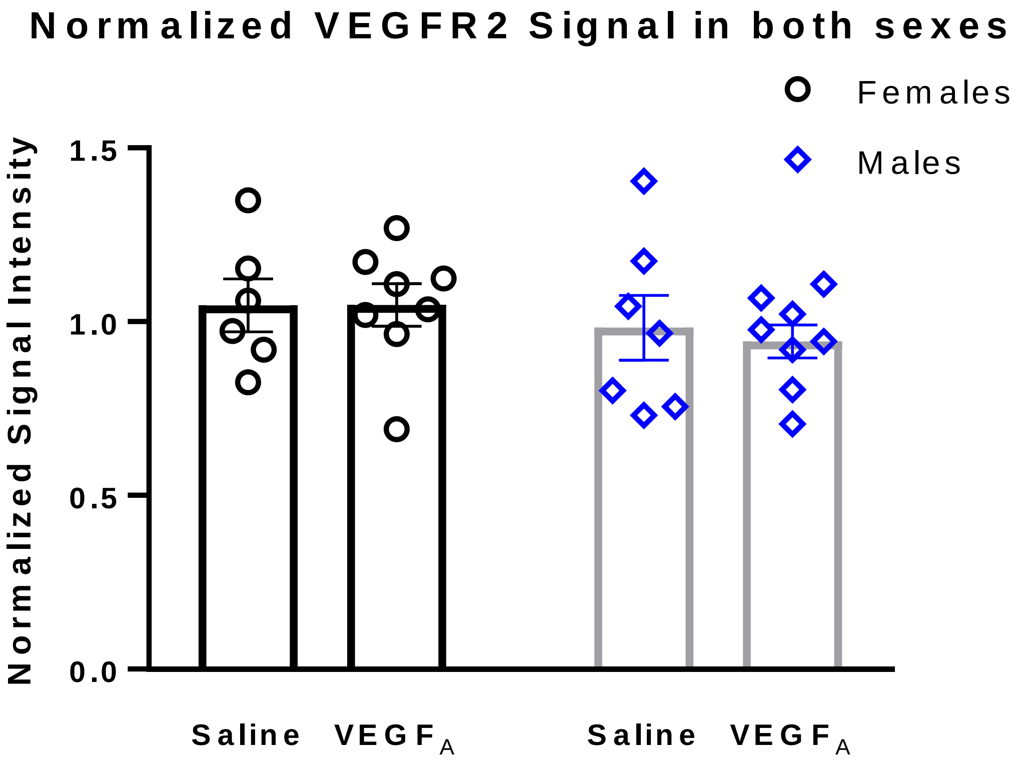

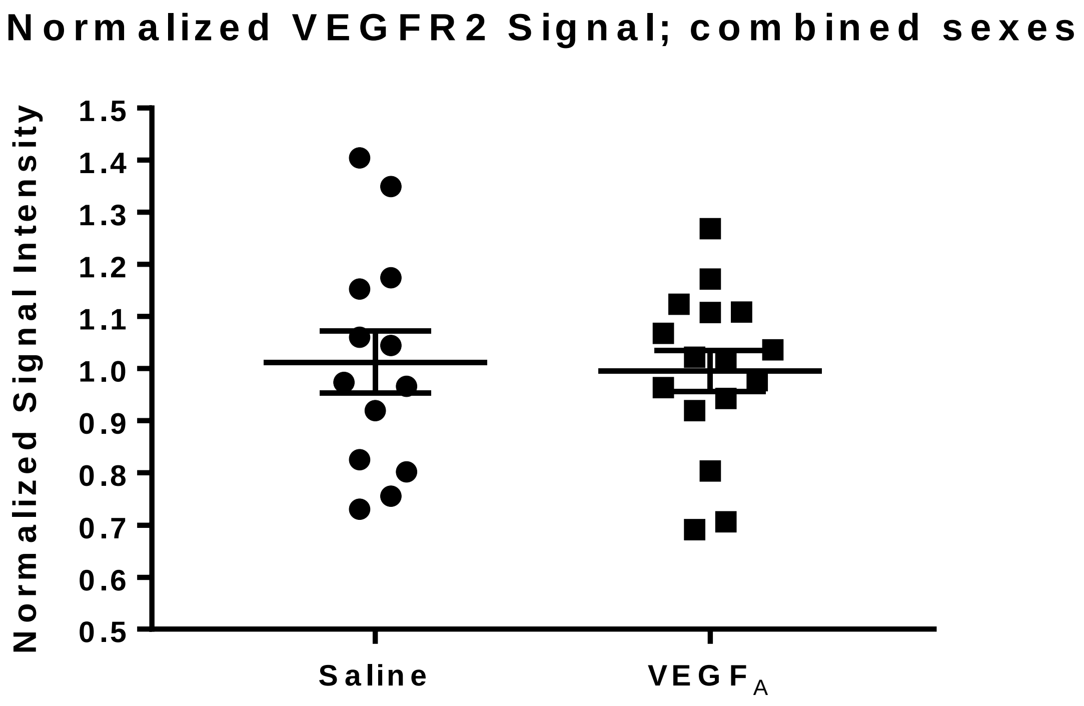

Total count	31	M	F
Saline	14	7	7
VEGF165	17	9	8

TRPV1 Staining Saline Instillation - 1

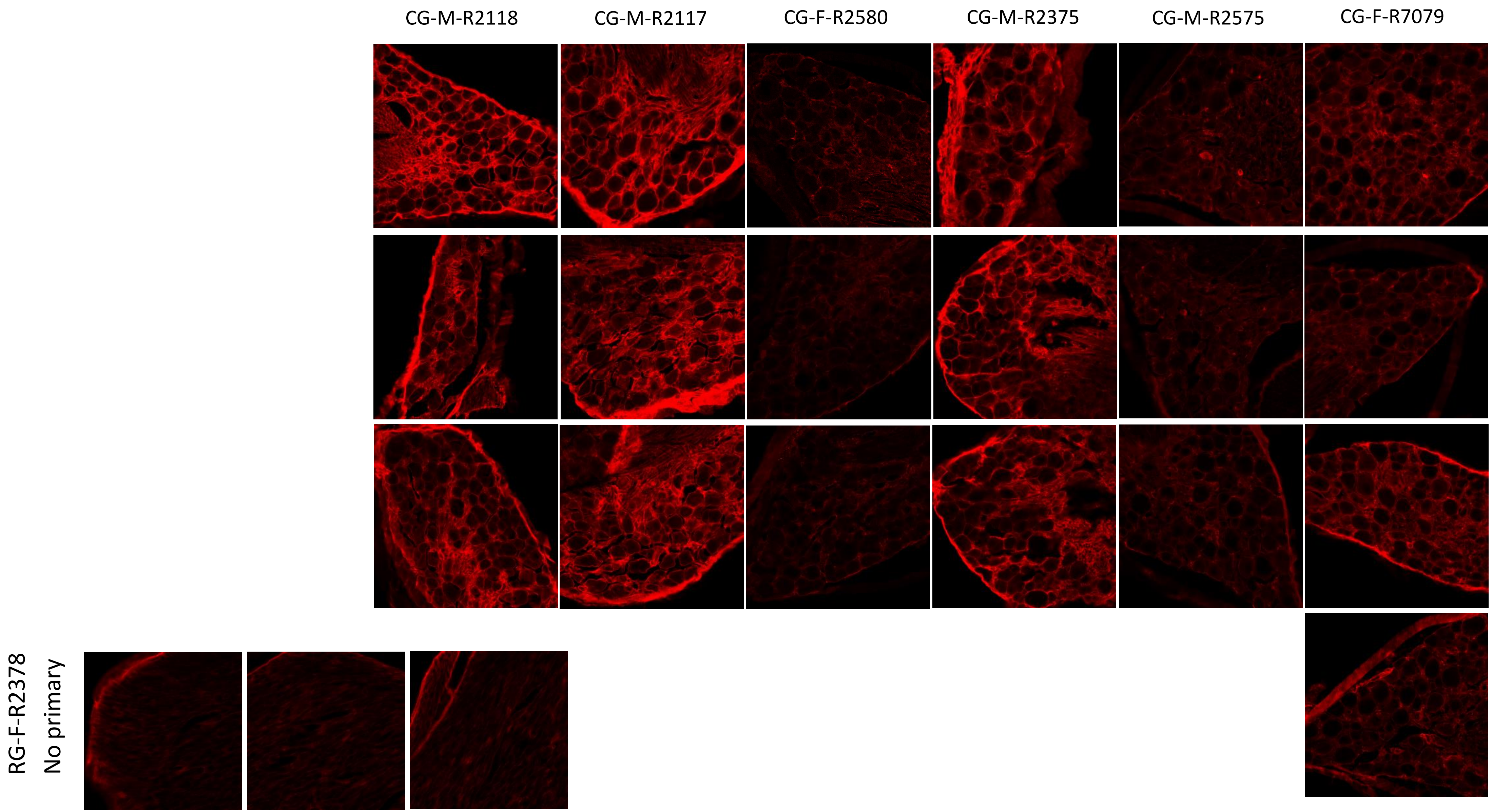

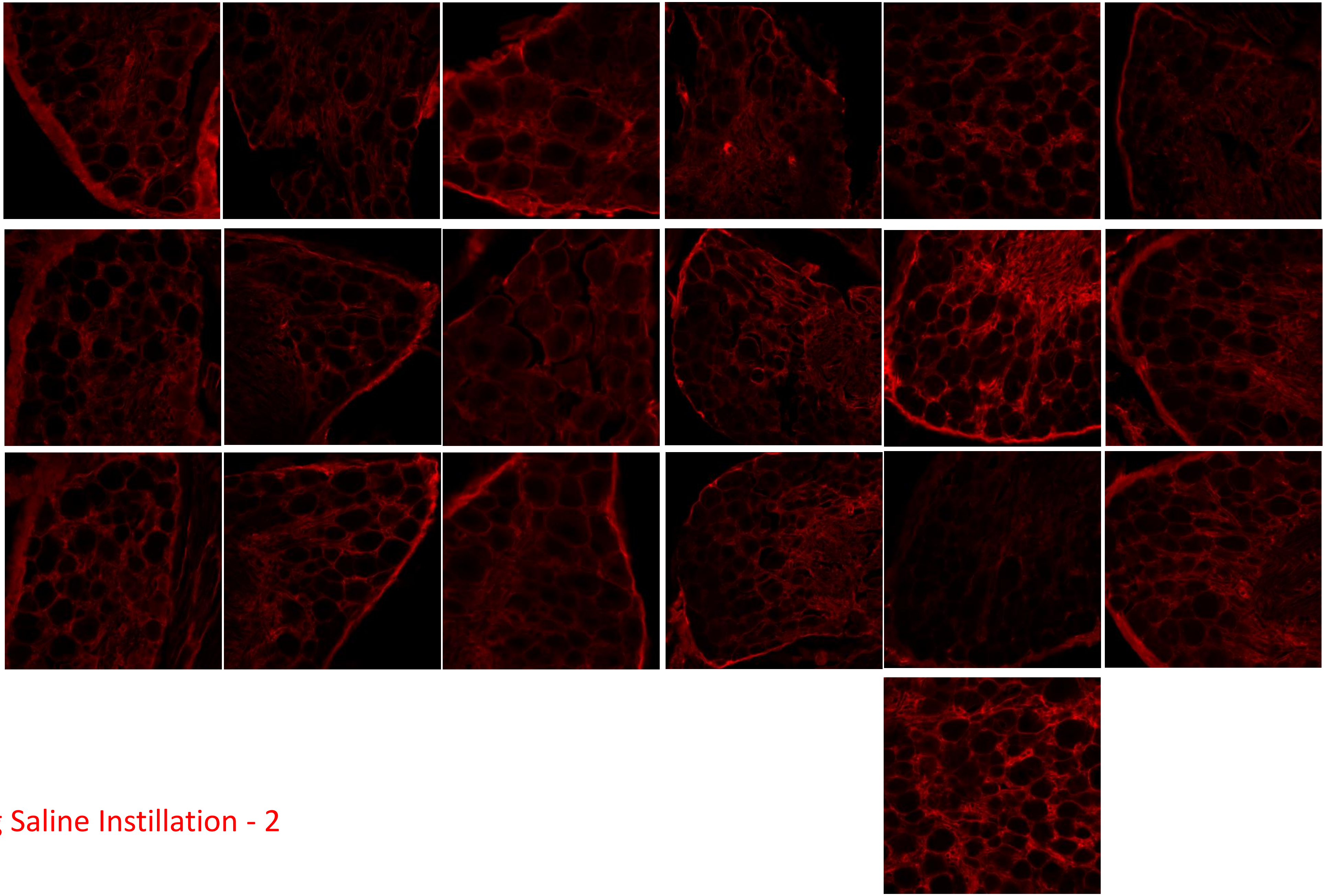

TRPV1 Staining Saline Instillation - 2

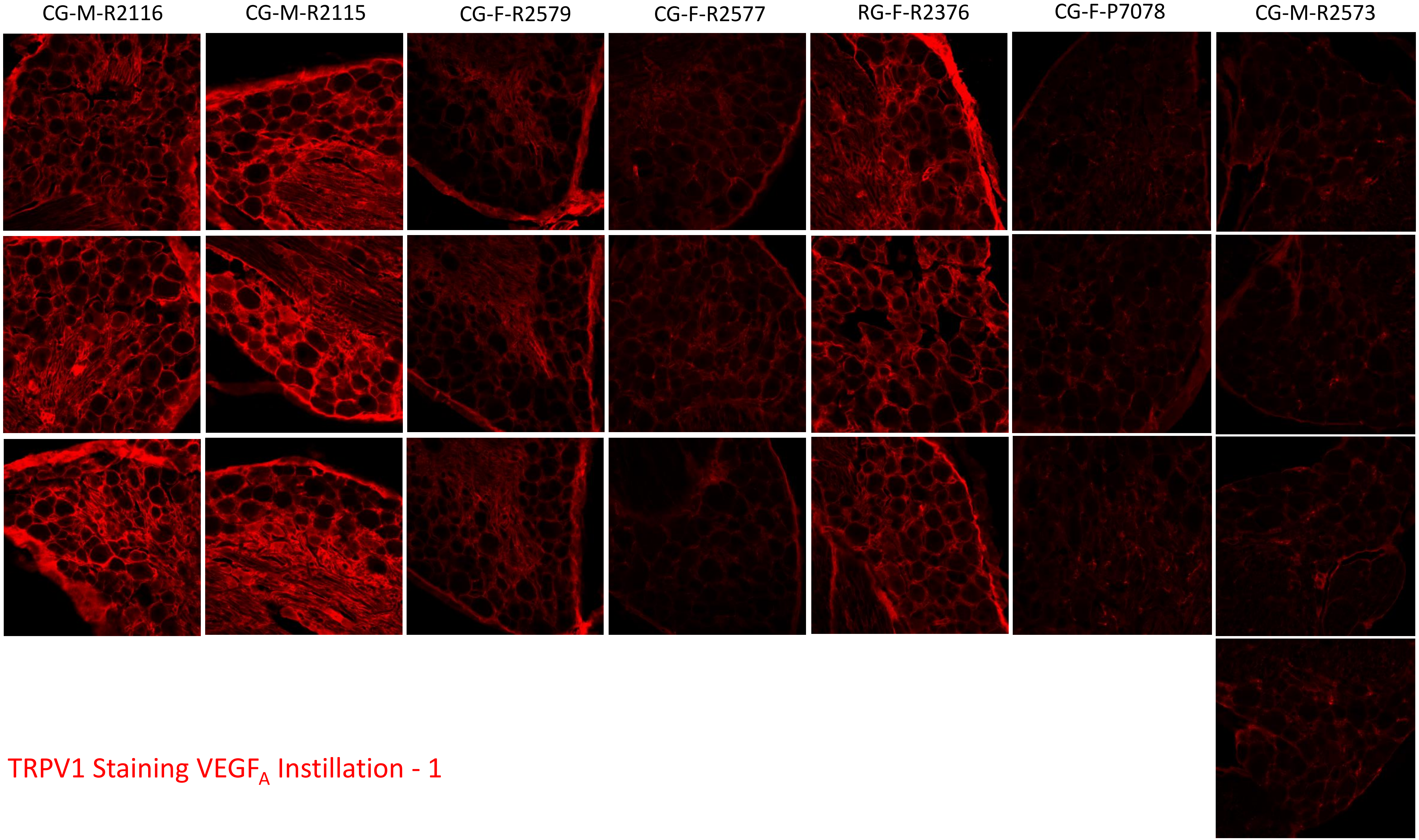

TRPV1 Staining $VEGF_A$ Instillation - 1

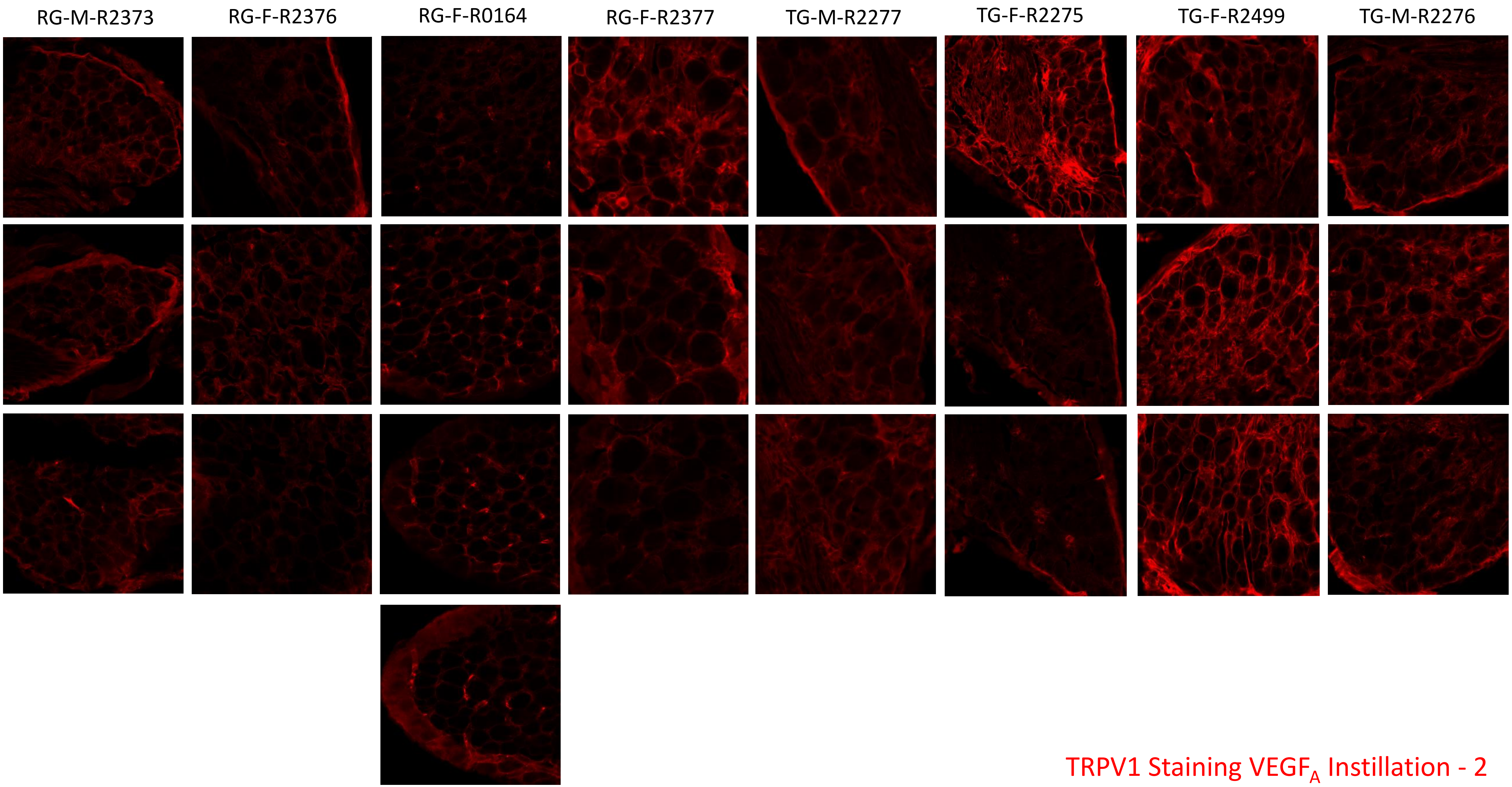

TRPV1 Staining $VEGF_A$ Instillation - 2

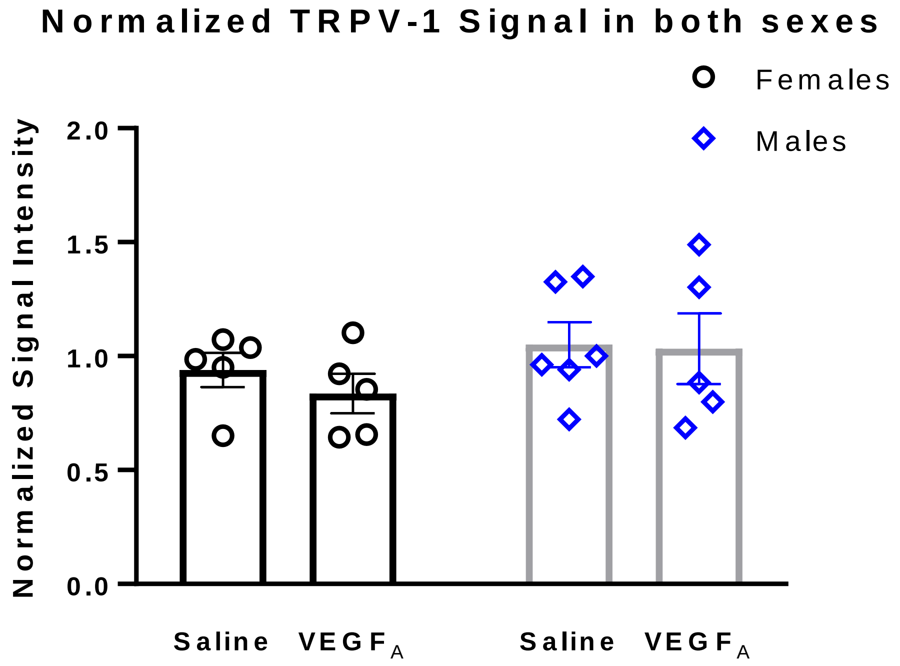

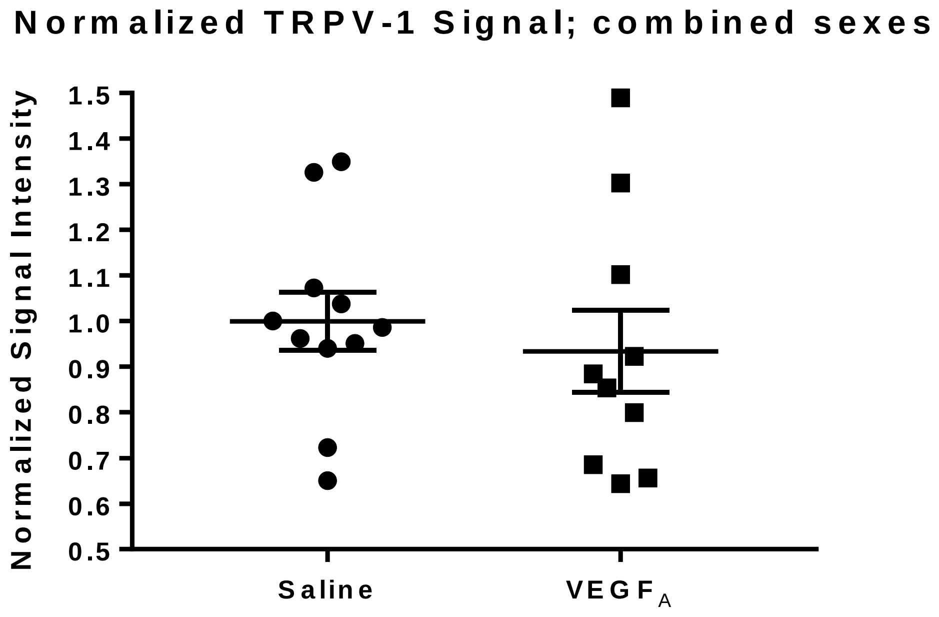

Total count	21	M	F
Saline	11	6	5
VEGF165	10	5	5

Antibody against TRPA1 exhibits signal in satellite glia cells and some DRG neurons.

TRPV1-ZsGreen +/+ mice (ZsGreen in 488) | **TRPA1** | **DAPI** | **Overlay**

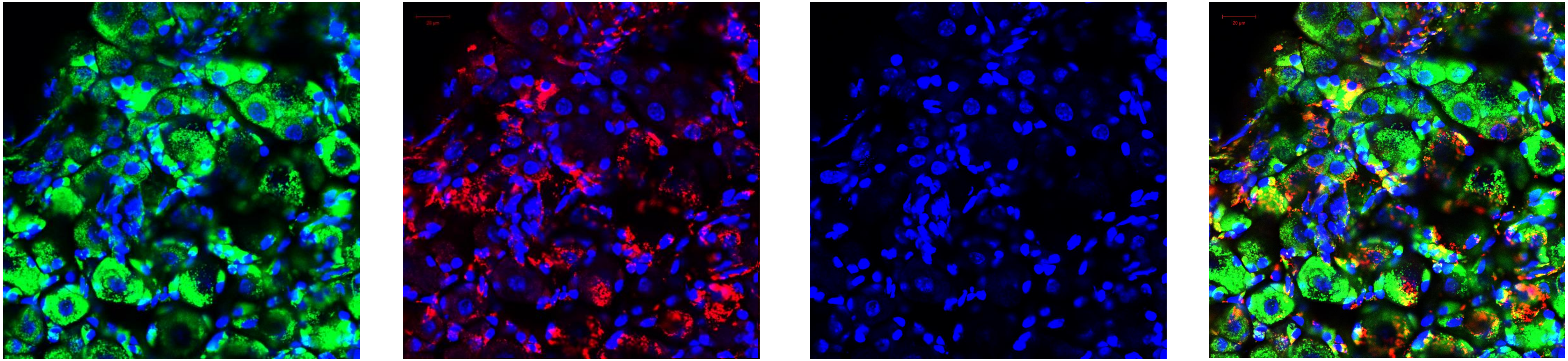

Antibody against TRPA1 exhibits signal in satellite glia cells and some DRG neurons.

TG VEGF R2275

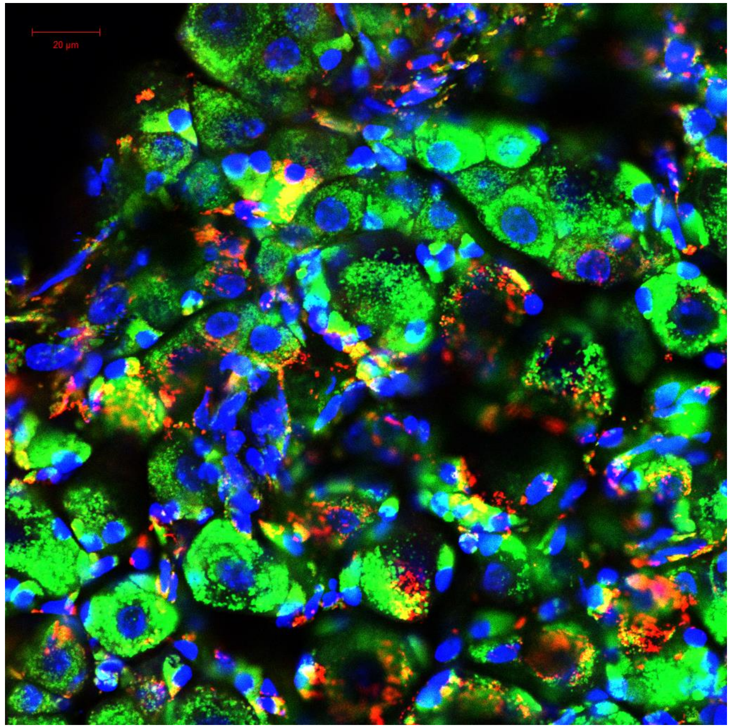

TG VEGF R2276

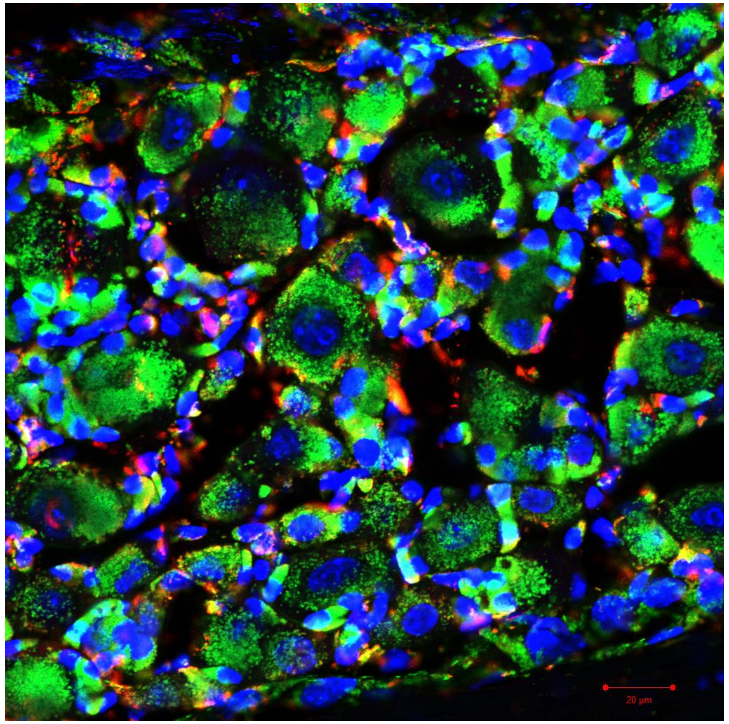

TG VEGF R2274 NP control

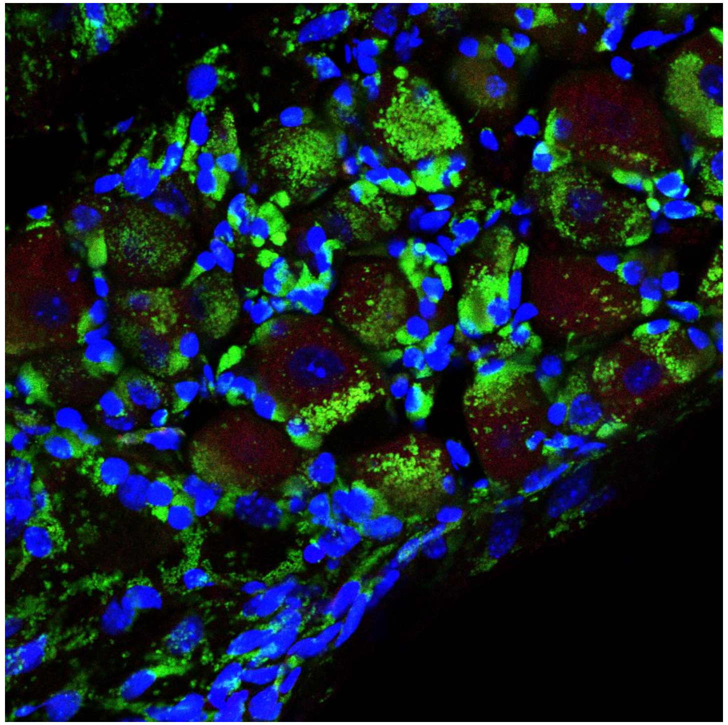

20190328 RGs/PBSVR1
RG female_R0165_40x_1
Saline Instillation

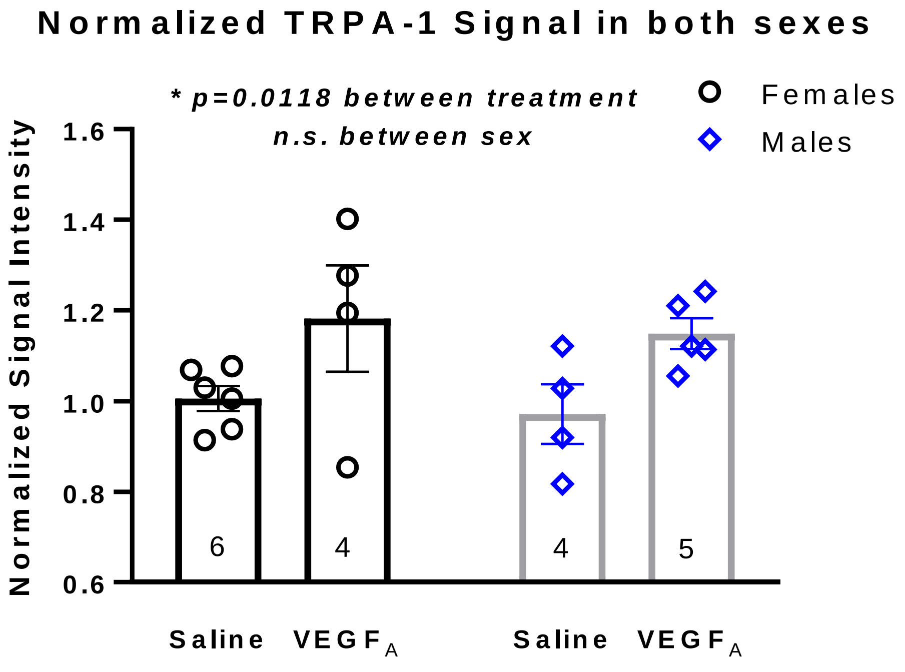

Normalized TRPA-1 Signal in both sexes
* p=0.0118 between treatment
n.s. between sex
Females
Males
Normalized Signal Intensity
1.6
1.4
1.2
1.0
0.8
0.6
6
4
4
5
Saline
VEGF A
Saline
VEGF A

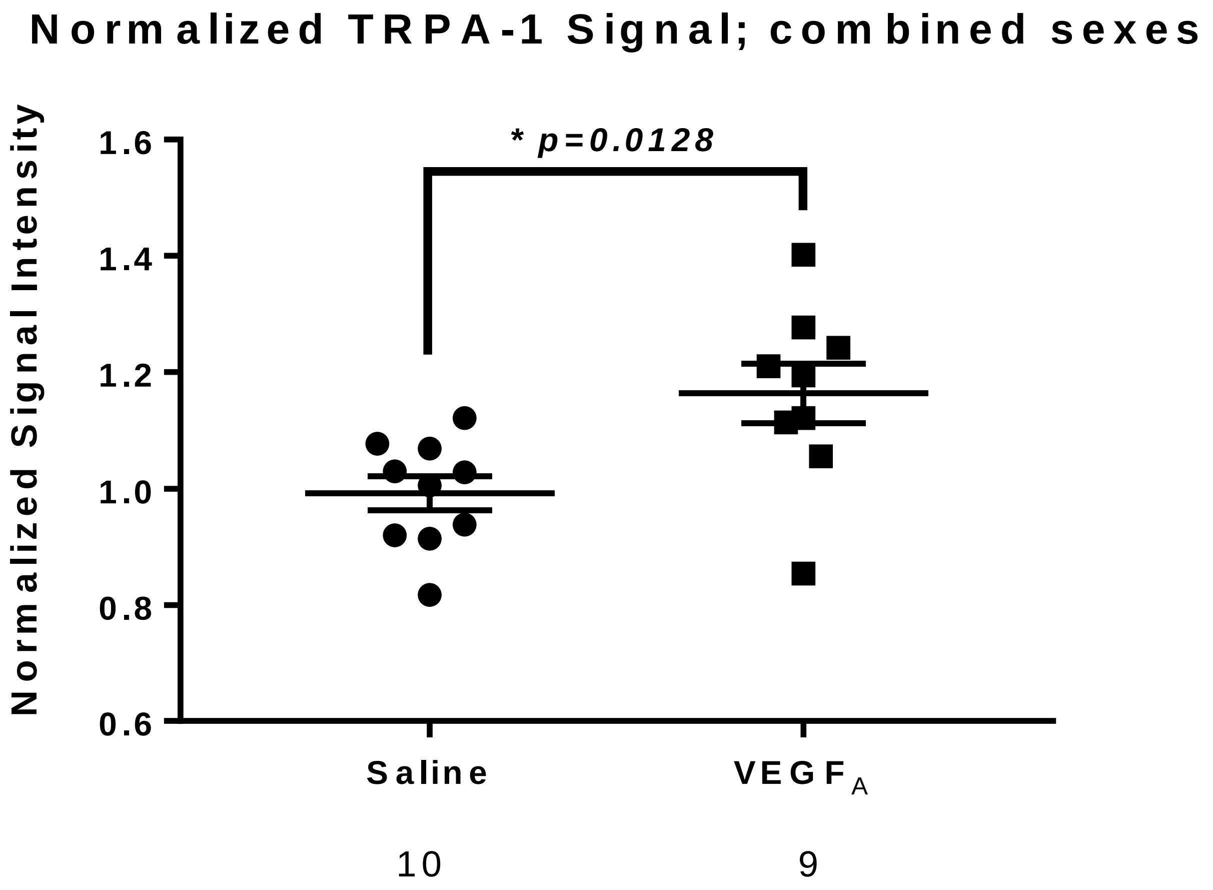

Normalized TRPA-1 Signal; combined sexes
* p=0.0128
Normalized Signal Intensity
1.6
1.4
1.2
1.0
0.8
0.6
Saline
VEGF A
10
9
